# CD8^+^ T Cells from a Novel T Cell Receptor Transgenic Mouse Induce Liver-Stage Immunity That Can Be Boosted by Blood-Stage Infection in Rodent Malaria

**DOI:** 10.1371/journal.ppat.1004135

**Published:** 2014-05-22

**Authors:** Lei Shong Lau, Daniel Fernandez-Ruiz, Vanessa Mollard, Angelika Sturm, Michelle A. Neller, Anton Cozijnsen, Julia L. Gregory, Gayle M. Davey, Claerwen M. Jones, Yi-Hsuan Lin, Ashraful Haque, Christian R. Engwerda, Catherine Q. Nie, Diana S. Hansen, Kenneth M. Murphy, Anthony T. Papenfuss, John J. Miles, Scott R. Burrows, Tania de Koning-Ward, Geoffrey I. McFadden, Francis R. Carbone, Brendan S. Crabb, William R. Heath

**Affiliations:** 1 Department of Microbiology and Immunology, The Peter Doherty Institute, University of Melbourne, Parkville, Victoria, Australia; 2 The School of Botany, University of Melbourne, Parkville, Victoria, Australia; 3 The QIMR Berghofer Medical Research Institute, Brisbane, Queensland, Australia; 4 The Walter and Eliza Hall Institute of Medical Research, Parkville, Victoria, Australia; 5 Macfarlane Burnet Institute for Medical Research & Public Health, Melbourne, Victoria, Australia; 6 Department of Pathology and Immunology, Washington University School of Medicine, St. Louis, Missouri, United States of America; 7 School of Medicine, The University of Queensland, Brisbane, Queensland, Australia; 8 Institute of Infection and Immunity, Cardiff University School of Medicine, Heath Park, Cardiff, Wales, United Kingdom; 9 School of Medicine, Deakin University, Waurn Ponds, Victoria, Australia; 10 Monash University, Clayton, Victoria, Australia; 11 The ARC Centre of Excellence in Advanced Molecular Imaging, University of Melbourne, Parkville, Australia; Faculdade de Medicina da Universidade de Lisboa, Portugal

## Abstract

To follow the fate of CD8^+^ T cells responsive to *Plasmodium berghei* ANKA (PbA) infection, we generated an MHC I-restricted TCR transgenic mouse line against this pathogen. T cells from this line, termed PbT-I T cells, were able to respond to blood-stage infection by PbA and two other rodent malaria species, *P. yoelii* XNL and *P. chabaudi* AS. These PbT-I T cells were also able to respond to sporozoites and to protect mice from liver-stage infection. Examination of the requirements for priming after intravenous administration of irradiated sporozoites, an effective vaccination approach, showed that the spleen rather than the liver was the main site of priming and that responses depended on CD8α^+^ dendritic cells. Importantly, sequential exposure to irradiated sporozoites followed two days later by blood-stage infection led to augmented PbT-I T cell expansion. These findings indicate that PbT-I T cells are a highly versatile tool for studying multiple stages and species of rodent malaria and suggest that cross-stage reactive CD8^+^ T cells may be utilized in liver-stage vaccine design to enable boosting by blood-stage infections.

## Author Summary

Malaria is a disease caused by *Plasmodium species*, which have a highly complex life cycle involving both liver and blood stages of mammalian infection. To prevent disease, one strategy has been to induce CD8^+^ T cells against liver-stage parasites, usually by immunization with stage-specific antigens. Here we describe a T cell receptor specificity that recognizes an antigen expressed in both the liver and blood stages of several rodent *Plasmodium species*. We generated a T cell receptor transgenic mouse with this specificity and showed that T cells from this line could protect against liver-stage infection. We used this novel tool to identify the site and cell-type involved in priming to a recently developed intravenous attenuated sporozoite vaccine shown to have efficacy in humans. We showed that CD8^+^ T cells with this specificity could protect against liver-stage infection while causing pathology to the blood stage. Finally, we provided evidence that T cells with cross-stage specificity can be primed and boosted on alternative stages, raising the possibility that antigens expressed in multiple stages might be ideal vaccine candidates for generating strong immunity to liver-stage parasites.

## Introduction

Malaria is a mosquito-transmitted disease found in a range of animals including man, non-human primates and rodents. It is caused by multiple *Plasmodium* species, several of which may infect the same animal species. For humans, the two most prevalent *Plasmodium* species are *P. falciparum* and *P. vivax*, with the former responsible for the bulk of lethal disease. Mice have been used as a convenient animal model for studying malaria, with three rodent *Plasmodium* species in use: (i) *P. chabaudi,* which can cause a disease that shows recrudescence and has many features in common with human malaria including anemia, sequestration of parasites, and metabolic acidosis [1]; (ii) *P. yoelii*, which has two very closely related strains that differ in their capacity to infect red blood cells and cause lethal disease [2]; and (iii) *P. berghei,* particularly the ANKA strain (PbA*)*, which has been used as a model for human cerebral malaria [3], [4], [5], a lethal complication of *P. falciparum* infection. While there is much debate as to the relevance of the PbA rodent infection model to human disease, the pathological processes underlying human cerebral malaria are relatively poorly characterized, making it difficult to accurately compare human and murine diseases. However, like human severe malaria, high parasite burden is required for multi-organ pathology in the PbA model [6], [7], [8]. In itself, the pathological process underlying experimental cerebral malaria (ECM) seen in PbA infections also offers insight into immune-mediated pathology in general, providing a rigorous experimental approach that can be easily manipulated to decipher various cellular and molecular contributions. In this rodent model, various cell types and cytokines have been reported to contribute to lethal ECM, with CD8^+^ T cells a major and essential contributor [9], [10], [11]. Infection with PbA leads to the activation of parasite-specific T cells that first expand in the spleen and then migrate to the brain, where they cause pathology [11]. Depletion of CD8^+^ T cells shortly before the onset of ECM prevents disease [11], supporting a role for these cells in the effector phase of disease pathology.

*Plasmodium* species have a complex life cycle with several distinct stages: a mosquito stage, from which sporozoites emerge to enter the mammalian hosts during a blood meal; a liver-stage where sporozoites enter hepatocytes and eventually develop into a large cohort of merozoites; and a blood stage, where merozoites are released into the blood and cause cyclic infection of erythrocytes. Disease symptoms and immune mediated pathology associated with malaria are limited to the blood-stage of infection, with the preceding liver stage being asymptomatic [12]. Despite this, sporozoite infection is not immunologically silent, with evidence that following pathogen entry via a mosquito bite, the immune response is initiated in the skin draining lymph nodes of mice [13], generating protective immunity that depends on CD8^+^ T cells and the cytokines TNFα and IFNγ [14]. Sporozoite-specific immunity can control infection in mice [15], non-human primates [16] and humans [17], [18], preventing development of blood-stage infection and its associated disease. As a consequence, researchers have explored the use of live sporozoites attenuated by irradiation or genetic engineering [19], [20], [21] or non-attenuated sporozoites controlled by drug curing, as potential approaches to vaccination [22]. Administration of irradiated cryopreserved sporozoites via the intravenous route was shown to provide superior immunity compared to cutaneous injection in non-human primates and mice [19]. More recently, vaccination of humans by the intravenous route demonstrated protection [21]. The success of the intravenous route was speculated to result from the direct access of parasites to the liver for development of immunity at this site. However, direct examination of where immunity was generated to this effective route of vaccination was not attempted.

During the different life-cycle stages, *Plasmodium* parasites adopt distinct morphologies and as a consequence express many stage-specific proteins, which are often the focus of immunity and vaccine design. However, many proteins are expressed throughout multiple stages of the life cycle [23] and in the mammalian host may be expected to contribute to immunity across multiple stages. While it has been suggested that blood-stage immunity may impair responses to liver-stage antigens [24], others have shown protection against liver-stage infection by prior blood-stage infection and cure [25], supporting the idea that antigens expressed at both stages may be capable of inducing protective immunity. However, direct demonstration of this capacity was not provided.

Here we describe the development of an MHC I-restricted, T cell receptor (TCR) transgenic murine line specific for PbA. We show that transgenic T cells from this line recognize an antigen expressed in both the blood-stage and the liver-stage of infection, demonstrating the potential for T cells with blood-stage-specificity to protect against sporozoite infection. T cells from this line detect a conserved antigen expressed by several rodent *Plasmodium* species including *P. chabaudi* and *P. yoelii,* rendering it a highly versatile immunological tool for dissecting CD8^+^ T cell immunity in malaria.

## Results

### Generation of an MHC I-restricted TCR transgenic mouse specific for PbA

**Figure 1:**
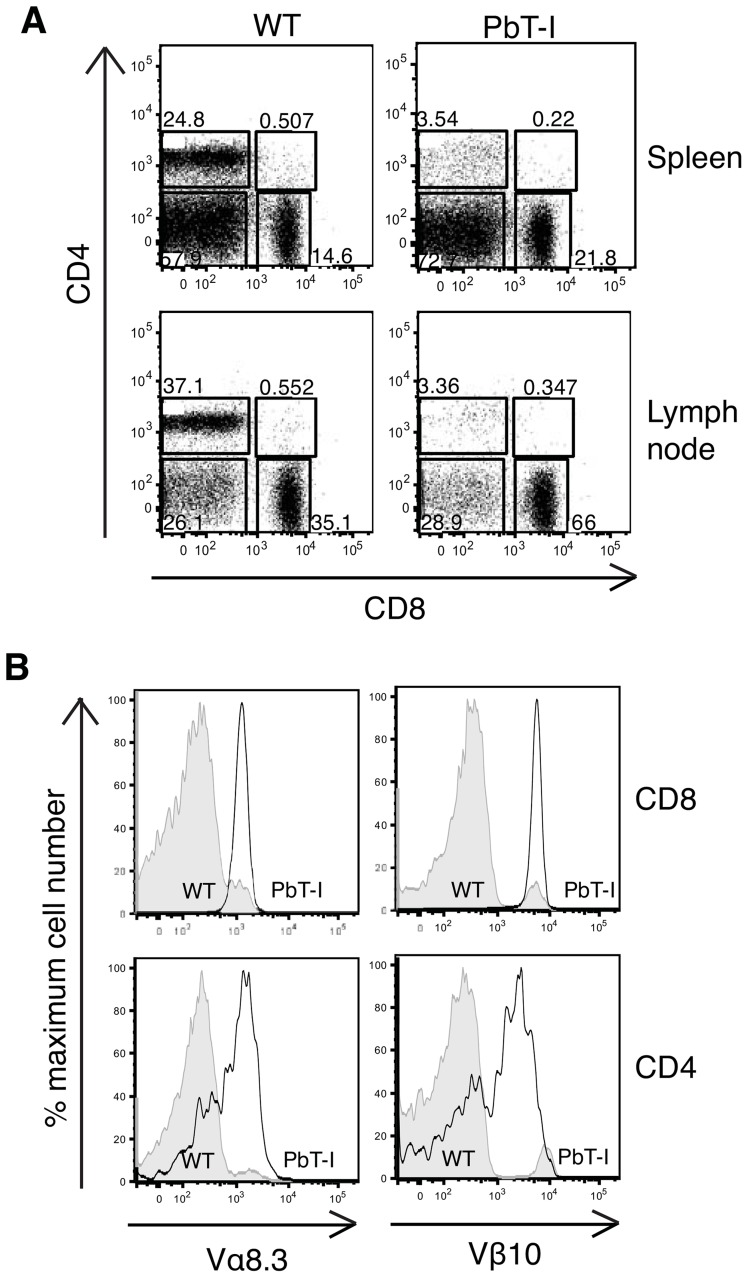
Characterization of T cells from the spleen and lymph node of PbT-I mice. Cells were harvested from the spleen and the lymph nodes of PbT-I transgenic or littermate control mice (WT). FACS analysis was performed to characterize the expression of CD8, CD4 and the transgenic TCR alpha (Vα8.3) and beta (Vβ10) chains. (A) Representative dot-plots showing the proportions of CD8 versus CD4 cells in the spleen and lymph node of PbT-I and WT mice. (B) Representative histograms showing the expression of the transgenic TCR Vα8.3 and Vβ10 chains on the CD8 or CD4 single-positive cells from the spleen. This experiment was repeated three times with two mice per experiment.

An MHC I-restricted TCR transgenic mouse line specific for blood-stage PbA (termed PbT-I) was generated using TCR genes isolated from a K^b^-restricted hybridoma termed B4 (**Figure S1**) originally derived from a T cell line isolated from a B6 mouse infected with blood-stage PbA. Analysis of spleen and lymph node (LN) cells from PbT-I mice showed a strong skewing towards CD8^+^ T cells (**Figure 1A**), with essentially all splenic (**Figure 1B**) and lymph node (**Figure S2**) CD8^+^ T cells expressing the Vα8.3 and Vβ10 transgenes. The few CD4^+^ T cells detected in the spleen and lymph node also expressed these transgenic receptors, though at a lower level indicative of co-expression of endogenous receptors. There was no reduction in spleen or lymph node cellularity relative to wild-type mice, with CD8^+^ T cells substituting for the lack of CD4^+^ T cells (**Figure S3**). Peripheral skewing towards CD8^+^ T cells was reflected in the thymus, where a large population of mature CD8^+^CD4^−^ T cells with high TCR expression was evident (**Figure S4**). In this case, total thymocyte numbers were reduced to about one third of wild-type (**Figure S3**), consistent with the cellularity of other TCR transgenic mice we have generated, and likely due to efficient positive selection [26].

### PbT-I cells respond to PbA *in vitro* and *in vivo*

To determine if PbT-I cells responded to blood-stage PbA, purified CD8^+^ T cells from PbT-I mice were labeled with CFSE and then stimulated *in vitro* with dendritic cells and lysate from either infected red blood cells (iRBC) of mixed stages or enriched as schizonts (**Figure S5**). This showed a dose-dependent proliferative response to both forms of antigen, though schizont lysate was more efficient.

**Figure 2:**
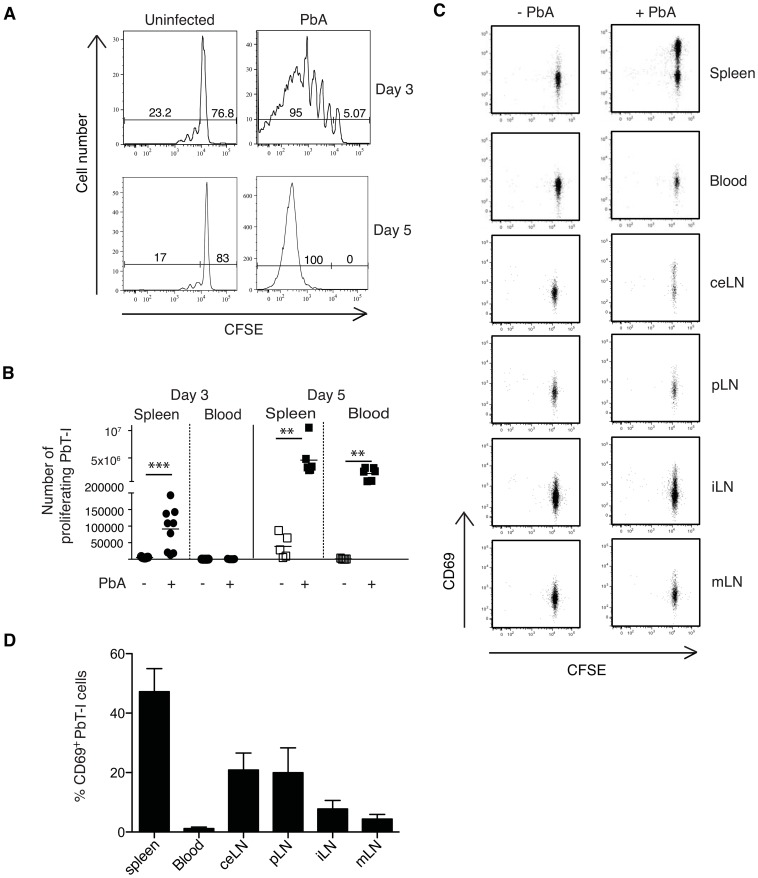
PbT-I cells respond in the spleen to i.v. blood-stage PbA. B6 mice were adoptively transferred with 2×10^6^ Ly5.1^+^ PbT-I cells and the next day infected i.v. with 10^6^ blood-stage PbA. Spleens were harvested three or five days later and the proliferation of PbT-I cells was analyzed. The gating strategy to identify PbT-I cells was similar to that shown in **Figure S13**. (A) Representative histograms showing the proliferation of PbT-I cells on day three or five post-infection. (B) Number of proliferating PbT-I cells in the spleen and blood of mice infected with PbA for three or five days. Data are pooled from three experiments. Each data point represents a mouse and the lines represent the mean. Data were compared using student t test (**, p<0.01; ***, p<0.001). (C) B6 mice were adoptively transferred with 10^6^ CFSE-labeled Ly5.1^+^ PbT-I cells. The next day, mice were injected i.v. with 10^6^ blood-stage PbA. Various tissues (spleen, blood, celiac lymph node (ceLN), portal LN (pLN), inguinal LN (iLN), mesenteric LN (mLN)) were harvested after 2 days and PbT-I cells examined for CD69 and CFSE expression. Profiles are gated on PbT-I cells. This experiment was performed three times (two-three mice per group) with similar results. Typical profiles are shown. (D) The mean percentage of CD69^+^ PbT-I cells for the analysis shown in (C). Histograms represent values from infected animals minus mean values from uninfected animals. Error bars represent standard error of the mean.

To test whether PbT-I cells also responded to PbA *in vivo*, PbT-I cells were labeled with CFSE and adoptively transferred into B6 mice one day before infection with blood-stage PbA. Three or 5 days later, mice were killed and the spleen and blood examined for proliferating PbT-I cells (**Figure 2A, B**). This revealed a vigorous response by PbT-I cells, which entered the blood from the spleen after day 3. The specificity of PbT-I cells for malarial antigen was demonstrated by their lack of response to intravenous (i.v.) infection with herpes simplex virus type I (HSV-1), an infection that efficiently stimulated viral glycoprotein B-specific transgenic T cells (gBT-I cells) in the same mice (**Figure S6**).

To more precisely determine where PbT-I cells were activated during the primary response to blood-stage PbA infection, B6 mice were injected with CFSE-labeled PbT-I cells one day before i.v. infection with blood-stage PbA, then various tissues were harvested 2 days later to examine expression of the early activation marker CD69 on PbT-I cells (**Figure 2C, D**). This showed that blood-stage infection caused T cell activation in the spleen, although some CD69 up-regulation was observed in liver-draining lymph nodes (portal and celiac LNs). Other lymph nodes showed no evidence of T cell activation.

To test whether PbT-I cells induced by blood-stage infection made cytokines and were able to degranulate, as required for lytic activity, mice were adoptively transferred with small numbers of GFP-expressing PbT-I cells and infected i.v. with blood-stage PbA. On day 8 post-infection, PbT-I cells were recovered from the spleen and briefly restimulated with anti-CD3 mAb to test for production of IFNγ, TNFα and CD107a, the latter of which is a surrogate marker for degranulation (**Figure S7**). This revealed that most PbT-I cells were able to produce both cytokines and degranulate.

### PbT-I cells cause experimental cerebral malaria

**Figure 3:**
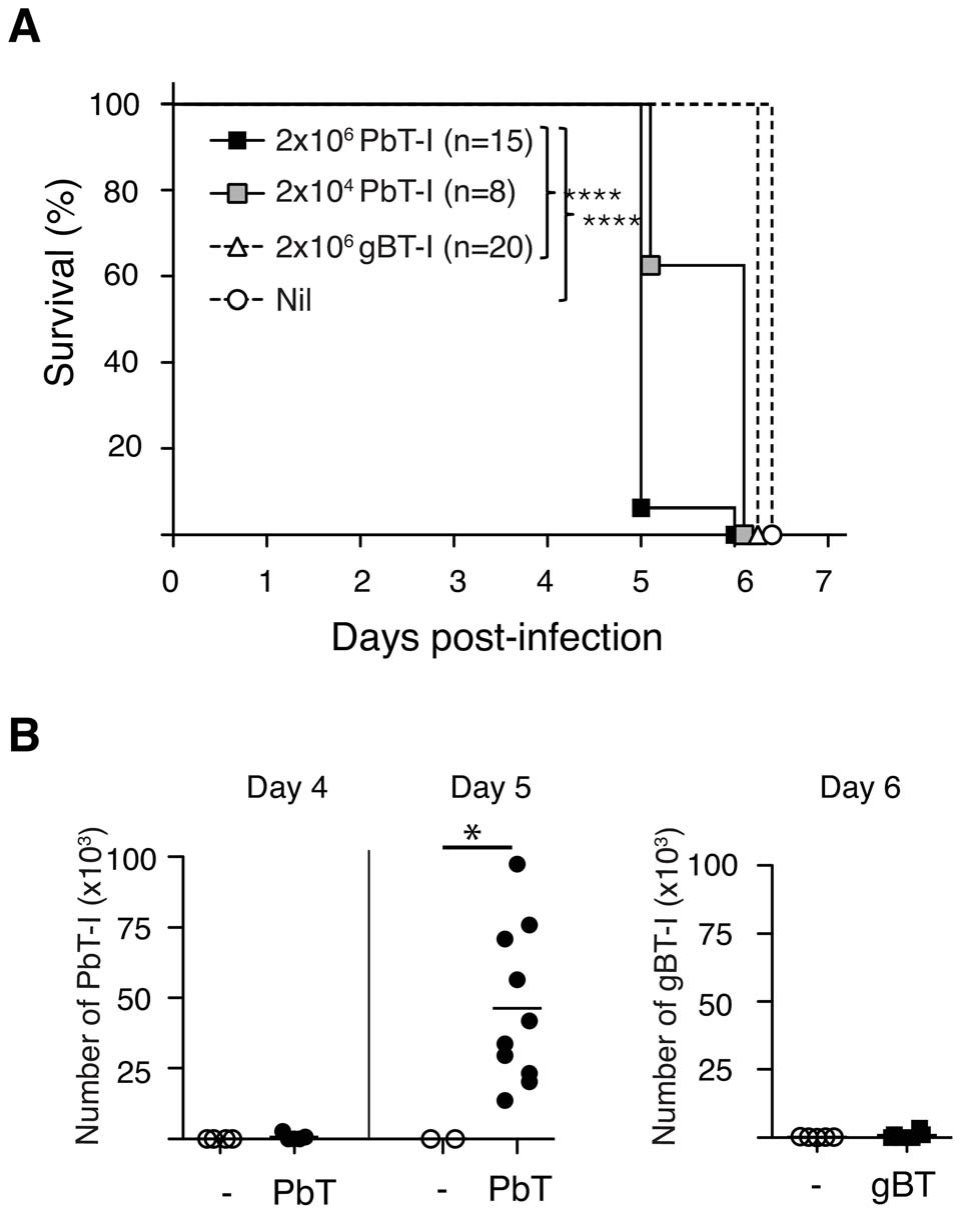
PbT-I cells infiltrate the brain and accelerate ECM. B6 mice were adoptively transferred with 2×10^6^ or 2×10^4^ Ly5.1^+^ PbT-I cells or 2×10^6^ herpes simplex virus-specific gBT-I cells or left uninjected. The next day mice were infected i.v. with 10^6^ blood-stage PbA. (A) Mice were monitored for the development of ECM. Data are pooled from three independent experiments. The differences between 2×10^6^ PbT-I and the group that did not receive any transgenic cells or the group that received gBT-I cells are statistically significant (p<0.0001) as determined by a Log-rank test. (B) Mice were adoptively transferred with PbT-I cells (filled circle) or gBT-I cells (filled square) or no cells (open circle) and were sacrificed on days 4, 5 or 6 post-infection. Their brains were then analysed for the infiltration of PbT-I cells (left) or gBT-I cells (right). Data are pooled from 2–4 experiments. Data were compared using student t test (*, p<0.05).

As CD8^+^ T cells have been implicated in the pathology of ECM, we asked whether transfer of PbT-I cells into B6 mice could accelerate this disease. B6 mice were injected with a high (2×10^6^) or low (2×10^4^) number of PbT-I cells or a high number of a herpes simplex virus-specific gBT-I cells, then infected with blood-stage PbA and monitored for disease (**Figure 3A**). This showed that PbT-I cells significantly accelerated disease onset, though only by about one day. ECM was accompanied by infiltration of PbT-I cells and endogenous CD8^+^ T cells, but not gBT-I cells into the brain of infected mice on days 5–6 post-infection (**Figure 3B** **and Figure S8**).

**Figure 4:**
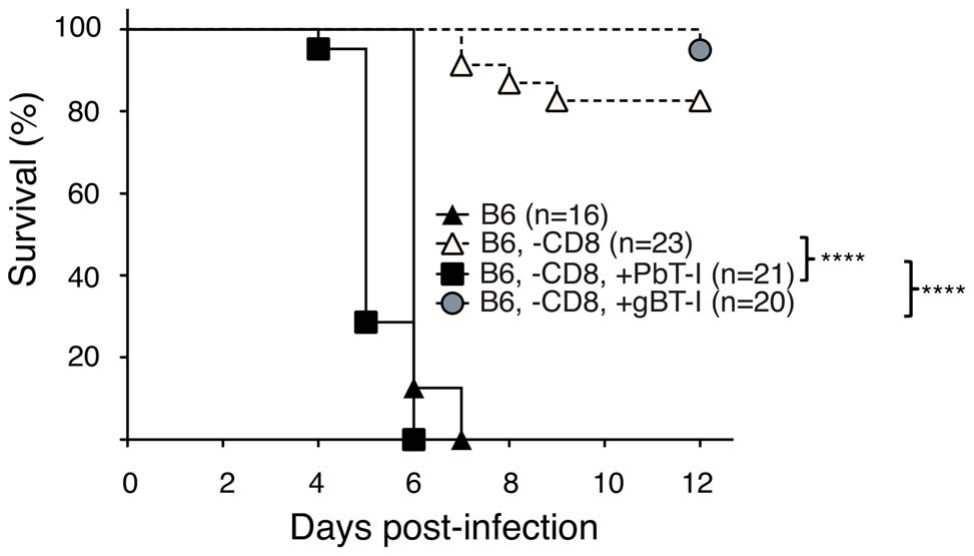
PbT-I cells induce ECM in mice lacking endogenous CD8^+^ T cells. B6 mice were either left undepleted (closed triangles) or were depleted of endogenous CD8^+^ T cells (-CD8) seven days before the adoptive transfer of 2×10^6^ naïve PbT-I (filled square) or gBT-I cells (filled circle) or no cells (open triangles). The next day, mice were infected i.v. with 10^6^ blood-stage PbA and monitored for the development of cerebral malaria. Data are pooled from three experiments. The difference in survival between the following groups was statistically significant (p<0.0001) as determined by the Log-Rank test: i. B6, -CD8, + PbT-I and B6, -CD8; ii. B6, -CD8, + PbT-I and B6, -CD8, + gBT-I.

To determine whether PbT-I cells could themselves cause ECM, endogenous CD8^+^ T cells were depleted from mice with anti-CD8 mAb and 7 days later replaced with PbT-I cells, control gBT-I cells or no T cells. One day later, these mice were infected with blood-stage PbA and examined for ECM onset. All mice given PbT-I cells developed ECM, while very few other CD8-depleted mice developed disease (**Figure 4**). Onset of ECM in a small fraction of the latter was likely due to incomplete depletion of endogenous CD8^+^ T cells in some mice. This could not be avoided because the dose of depleting anti-CD8 antibody had to be sufficient to deplete virtually all endogenous CD8^+^ T cells while leaving little antibody to persist until adoptively transfer of PbT-I cells a week later (otherwise remaining anti-CD8 mAb would have depleted these PbT-I cells). H&E staining of the brains of mice that received PbT-I cells showed typical features of CM, such as haemorrhages and intravascular accumulation of RBC and leukocytes (**Figure S9**). These data clearly showed that PbT-I cells were able to cause ECM.

### PbT-I cells cross-react on other species of *Plasmodium*

**Figure 5:**
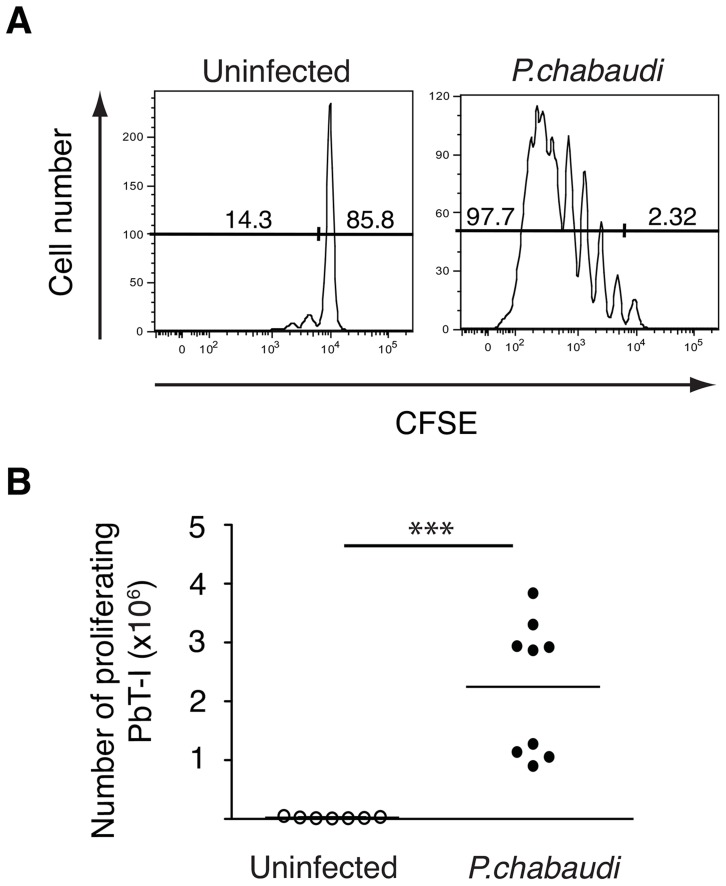
PbT-I cells respond to *P. chabaudi* AS. B6 mice were adoptively transferred i.v. with 2×10^6^ CFSE-labeled PbT-I cells. The next day, mice were injected i.v. with 10^5^ *P. chabaudi* AS. Six or seven days later, spleens were harvested and the proliferation of PbT-I was analyzed. (A) Representative histograms of CFSE-labeled PbT-I cells in the spleen of uninfected or *P. chabaudi* infected mice. (B) Number of PbT-I cells in the spleens of mice on days 6–7. The lines represent the mean and each data point represents a mouse. Data are pooled from three experiments. Data were compared using student t test (***, p<0.001).

As the precise specificity of PbT-I cells was unknown, we determined whether they recognized other species of *Plasmodium.* CFSE-labeled PbT-I cells were adoptively transferred into B6 mice that were then infected with blood-stage *P. chabaudi* AS; 6 or 7 days later proliferation of PbT-I cells was assessed in the spleen (**Figure 5**). This showed that PbT-I cells could proliferate in response to blood-stage *P. chabaudi* AS. In a similar set of experiments, PbT-I cells were also shown to respond to blood-stage infection with *P. yoelii* XNL (**Figure S10**). These findings indicated that PbT-I cells have specificity for multiple *Plasmodium* species that cause rodent malaria.

### PbT-I cells respond to irradiated PbA sporozoites

**Figure 6:**
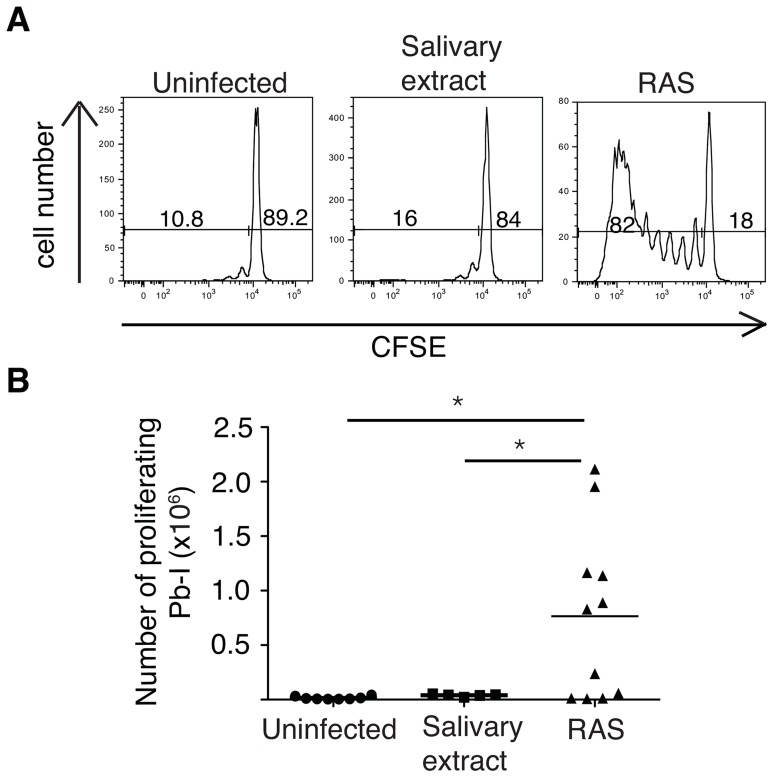
PbT-I cells proliferate in response to irradiated sporozoites. B6 mice were adoptively transferred with 2×10^6^ CFSE-labeled PbT-I cells. The next day, mice were injected i.v. with 5×10^4^–10^5^ radiation attenuated sporozoites (RAS) or equivalent salivary extract. Four days later spleens were harvested and the proliferation of PbT-I cells was analyzed. (A) Representative histograms showing the proliferation of PbT-I cells in response to irradiated sporozoites. (B) Pooled data showing the number of divided PbT-I cells from four experiments. The lines represent the mean and each data point represents a mouse. Mice similarly treated but left until day 7 post-challenge showed no breakthrough in blood-stage infection indicating full attenuation of sporozoites. Data were compared by one-way ANOVA and Tukey's multiple comparison test (*, p<0.05).

While our PbT-I line was generated to blood-stage parasite infection, a proportion of antigens expressed in the blood stage are also expressed by sporozoites and during the liver-stage of infection [23]. To address whether sporozoites could stimulate PbT-I cells, we adoptively transferred CFSE-labeled PbT-I cells into B6 mice and then injected them i.v. with radiation-attenuated PbA sporozoites (RAS). On day 4 post-infection, proliferating PbT-I cells were detected in the spleen indicating their capacity to respond to sporozoites (**Figure 6**). Additional mice examined on day 7 did not progress to patency, indicating that day 4 responses were induced by sporozoites and not by break-through blood-stage parasites. Eight days after infection, PbT-I cells harvested from the spleen produced IFNγ, TNFα and CD107a (**Figure S11**), indicating their development of effector function.

### Proliferation of PbT-I cells to i.v. sporozoites occurs mainly in the spleen and depends on CD8α^+^ DC

**Figure 7:**
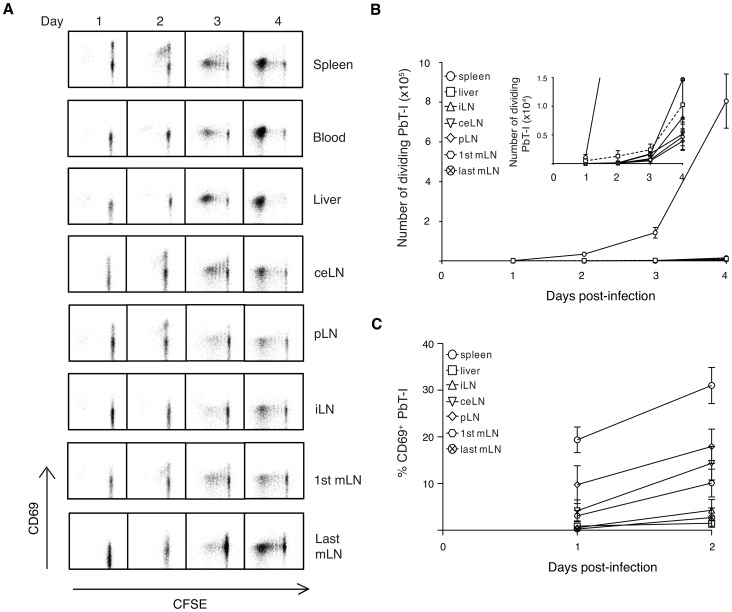
PbT-I cells are primed mainly in the spleen when irradiated sporozoites are delivered by the i.v. route. B6 mice were adoptively transferred with 10^6^ CFSE-labeled PbT-I cells. The next day, mice were injected i.v. with 10^5^ irradiated PbA sporozoites. Various organs (spleen, blood, liver, different lymph nodes) were harvested on days 1–4 post-vaccination and the activation and proliferation of PbT-I cells was analyzed by flow cytometry. For gating strategy to identify PbT-I cells see **Figure S13**. (A) Representative histograms showing the proliferation of PbT-I cells versus the upregulation of CD69 in the various organs. (B) Number of divided PbT-I cells in the different organs. The insert shows a more sensitive scale to identify the few divided PbT-I cells in the liver and lymph nodes on days three and four post-infection. (C) Percentage of CD69^+^ PbT-I cells in each organ on days 1-2. Error bars represent standard error of the mean. Data are pooled from three independent experiments.

A recent report suggested that the efficiency of intravenous vaccination with irradiated sporozoites relative to subcutaneous vaccination may be because the former route allows more parasites to reach the liver for priming of protective immunity [19]. To test whether priming by irradiate parasites occurred in the liver, we injected irradiated sporozoites intravenously and then 1–4 days later examined the activation (CD69 expression) (**Figure 7A, C**) and proliferation (**Figure 7A, B**) of PbT-I cells in the liver and various lymphoid tissues including the spleen and lymph nodes. Upregulation of CD69 was seen as early as one day after infection and was primarily detected in the spleen, with some expression also seen in the liver draining lymph nodes (celiac LN, portal LN and the 1^st^ mesenteric LN) [27]. Proliferation closely followed on day 2, almost entirely in the spleen. These data suggested that PbT-I cells responded to sporozoites by CD69 upregulation and extensive initial proliferation in the spleen and to a lesser extent in the liver-draining lymph nodes, but not in the liver nor other lymph nodes. Divided cells were only evident in the liver once they were present in the blood and had divided extensively, suggesting initiation of proliferation elsewhere, most likely in the spleen.

**Figure 8:**
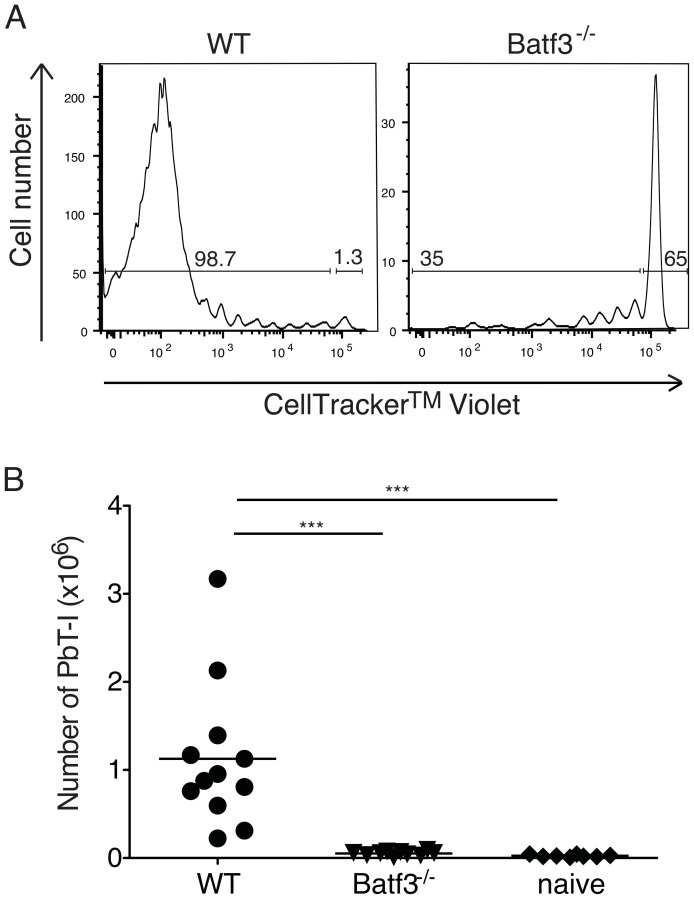
CD8α^+^ DC are required for presentation of irradiated sporozoites delivered by the i.v. route. B6 or Batf3^-/-^ mice were adoptively transferred with 10^6^ CellTracker Violet-labeled PbT-I cells then infected with irradiated PbA sporozoites. (A) Representative histograms show the proliferation of PbT-I cells in B6 and Batf3^-/-^ mice on day 5 after injection with irradiated sporozoites. (B) Pooled data showing the proliferation of PbT-I cells from three experiments. The lines represent the mean and each data point represents a mouse. Data were compared by one-way ANOVA and Tukey's multiple comparison test (***, p<0.001).

CD8α^+^ DC are critical for generating immunity to blood-stage infection [28], [29] and recently the human DC subset equivalent, BDCA3^+^ DC, have been implicated in severe malaria in humans [30], [31]. To address whether CD8α^+^ DC also participated in responses to sporozoites, we examined proliferation of PbT-I cells in Batf3^-/-^ mice, which lack this DC subset (**Figure 8**). The poor proliferation in Batf3^-/-^ mice compared to wild-type mice revealed that this response was dependent on CD8α^+^ dendritic cells.

### Cumulative expansion of PbT-I cells responding to liver- and blood-stage infection

**Figure 9:**
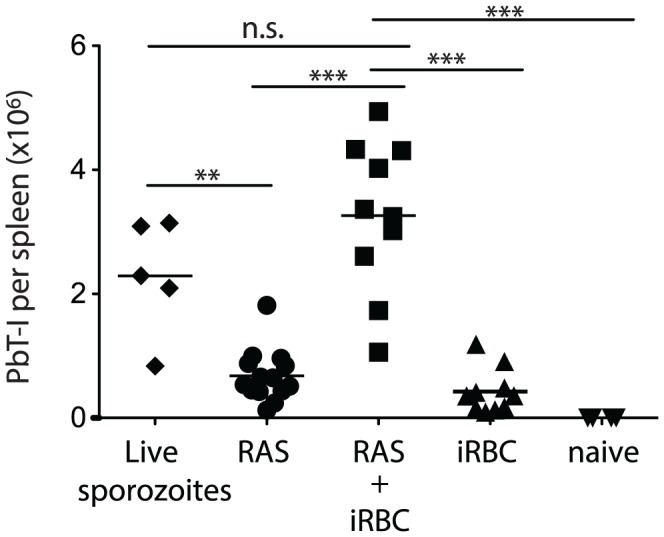
PbT-I cells proliferate to greater numbers when sequentially primed with liver-stage then blood-stage PbA parasites. B6 mice were adoptively transferred with 5×10^4^ GFP-expressing PbT-I cells then on day 0 they were injected with 5×10^4^ live sporozoites alone, or 5×10^4^ radiation-attenuated sporozoites alone (RAS), or RAS followed 2 days later by 10^4^ iRBC (RAS + iRBC), or nothing followed 2 days later by 10^4^ iRBC (iRBC) or left uninfected (naïve). On day 7 spleens were harvested and the number of PbT-I cells enumerated. Data are pooled from four experiments. At the time of sacrifice, blood parasitemias were equivalent in the two groups of mice given iRBC (RAS + iRBC  =  5.6±1.8; iRBC alone  =  5.1±0.7). Data were compared by one-way ANOVA and Tukey's multiple comparison test (n.s., non-significant; **, p<0.01; ***, p<0.001).

It has been reported that blood-stage infection can impair immunity to liver-stage antigens [24], though this is disputed by evidence that there is an equivalent response by liver-stage-specific transgenic T cells to sporozoites in the presence or absence of a subsequent blood-stage infection [32]. To resolve this issue with respect to CD8^+^ T cell-mediated immunity, we examined the expansion of PbT-I cells after exposing mice to live sporozoites (which will infect the liver then generate blood-stage infection), or irradiated sporozoites alone or followed by blood-stage (iRBC) infection 2 days later, mimicking the time for blood-stage egress after live sporozoite infection (**Figure 9**). Our results clearly showed that naïve PbT-I cells proliferated to reach greater numbers if additionally exposed to blood-stage infection, indicating that T cells with cross-stage specificity can show cumulative expansion to the liver and bloods stages.

Since sporozoite antigen has been shown to persist in other models [33], and we could demonstrate some proliferation of PbT-I cells transferred 2 days but not 7 days after injection of irradiated sporozoites (**Figure S12A, B**), indicating at least short-term persistence of the PbT-I antigen, it remained possible that augmented proliferation of PbT-I cells due to blood-stage infection might simply relate to additional inflammatory effects, rather than provision of antigen. To test whether inflammation alone could boost PbT-I expansion to irradiated sporozoites, 20 nmol of 1668 CpG oligonucleotide (CpG) was used as an inflammatory signal on day 2 and its effect on expansion of PbT-I cells examined (**Figure S12C**). CpG-mediated inflammation failed to induce a significant increase in PbT-I cell numbers in mice given irradiated sporozoites two days earlier, suggesting that antigen provided by blood-stage infection may be important for enhanced proliferation. This did not, however, formally excluding a role for inflammatory signals distinct from CpG that are associated with blood-stage infection.

### PbT-I cells protect B6 mice from sporozoite infection

**Figure 10:**
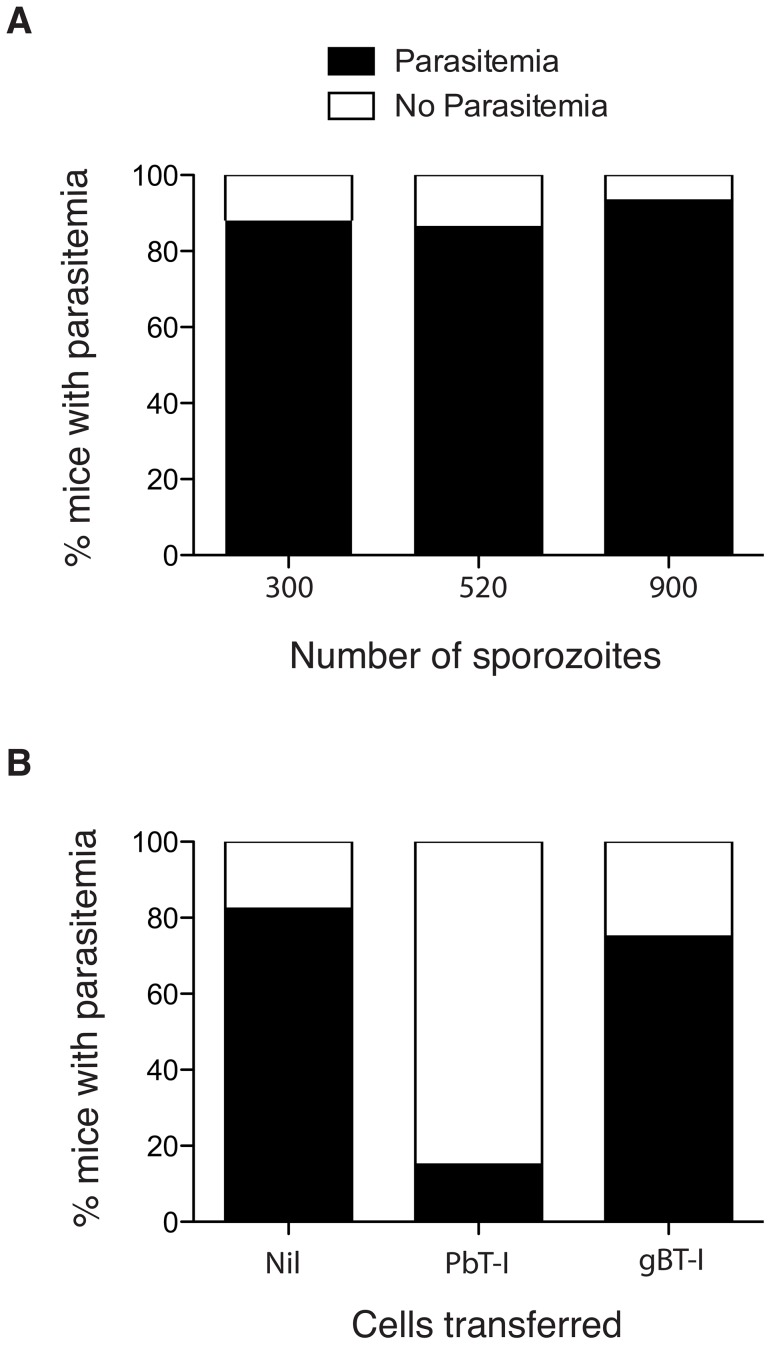
Activated PbT-I cells confer protection against a sporozoite challenge. (A) B6 mice were infected i.v. with 300 (n = 25), 520 (n = 22) or 900 (n = 15) PbA sporozoites and the % of mice that developed a blood-stage infection (black bars) by day 14 is shown. Data are pooled from three to five experiments. Data were analyzed using Fisher's exact test with no significant difference between groups (p>0.05). (B) B6 mice were adoptively transferred with 7×10^6^ *in vitro* activated PbT-I cells (n = 20) or activated virus-specific gBT-I cells (n = 16) or Nil (n = 17). Two hours later, mice were infected i.v. with 520 sporozoites. Blood-stage parasitemia was monitored to day 14 post-infection. Shown is the percentage of mice that develop a blood-stage infection (black bars) by day 14. Data are pooled from three experiments. Data were analyzed using Fisher's exact test with the PbT-I treated group significantly different from the other two groups (p<0.0001) and no significant difference between these two groups (p>0.05).

Because only one parasitized hepatocyte needs to survive to deploy thousands of merozoites into the blood and seed blood-stage infection, it is very difficult to prevent malaria with vaccines directed at pre-erythrocytic stages. It follows that any vaccine targeting pre-erythrocytic stages of infection must generate sterile immunity to be effective. As the antigen recognized by PbT-I cells was expressed by sporozoites, we asked whether this antigen might represent a vaccine candidate capable of eliciting sterile hepatic immunity. To assess this, we asked whether PbT-I cells could provide protective immunity to liver-stage infection. First, we determined an infectious dose of sporozoites that would lead to just under 100% blood-stage infection in the absence of PbT-I cells (**Figure 10A**). From this we chose 520 sporozoites as our infectious dose. To test the protective capacity of PbT-I cells, these cells or control virus-specific gBT-I cells were activated *in vitro* and then 7×10^6^ cells adoptively transferred into naïve B6 mice that were subsequently challenged with 520 live sporozoites (**Figure 10B**). By monitoring these mice for blood parasitemia, we showed that PbT-I cells, but not gBT-I cells, could prevent progression to blood-stage infection, protecting mice from infection. This indicated that the antigen recognized by PbT-I cells has the potential to generate sterilizing immunity to liver-stage infection.

### Identification of a candidate antigen recognized by PbT-I cells

**Figure 11:**
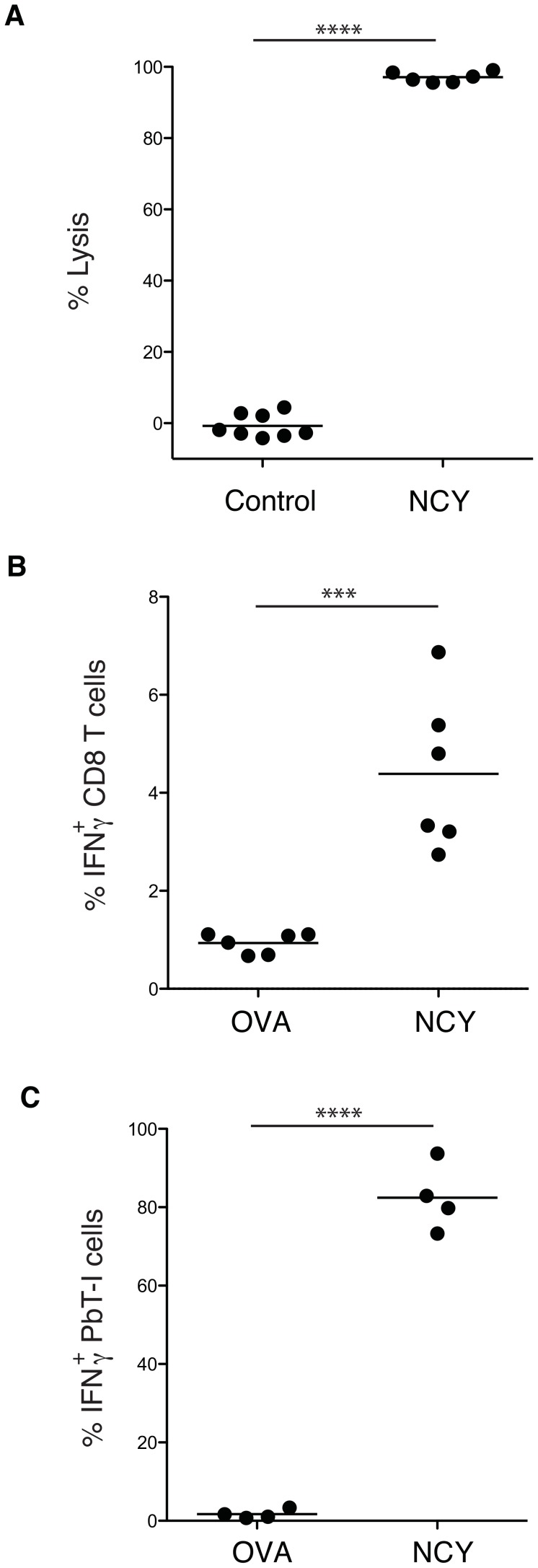
Identification of a peptide antigen (NCYDFNNI) recognized by endogenous T cells and PbT-I cells from PbA-infected mice. (A) B6 mice were infected with 10^6^ blood-stage PbA then cured by treatment with chloroquine from days 4 to 6. On day 7 mice were adoptively transferred with peptide-labeled target cells and a day later spleens were recovered and examined for target cell killing. Data are from two experiments, each of which used a test peptide, NCYDFNNI, and control peptides (NNFDFNNL or NIYDFNFI; pooled). (B, C) IFNγ production by T cells responding to PbA. B6 mice were either left untreated (B) or were adoptively transferred with 5×10^4^ PbT-I cells (C). All mice were then infected with 10^6^ blood-stage PbA and cured of infection by chloroquine treatment from days 4 to 6. On day 7, spleens were removed and an intracellular cytokine assay performed to detect IFNγ expression by endogenous CD8 T cells (B) or PbT-I cells (C). Data are from two experiments, each of which used a test peptide, NCYDFNNI, and a control peptide (SIINFEKL; OVA). Data were compared using student t test (***, p<0.001; ****, p<0.0001).

To identify the antigenic determinant recognized by PbT-I cells, we used an octamer combinatorial peptide library scan [34] to identify amino acid residues important for PbT-I activation as measured by MIP1β production (**data not shown**). These residues were then used to generate a octamer motif (x-x-x-(CD)-(WF)-N-x-(LMIV); where x is any amino acid and residues in brackets are valid for that position) to search the genomes of the three rodent malaria species for which PbT-I cells showed reactivity. 151 peptides fitting this motif were then examined for their capacity to stimulate PbT-I cells either by CD69 expression or MIP1β production (**data not shown**). Six peptide sequences caused some T cell activation but only one of these (NCYDFNNI (NCY)) was found to act as a target antigen for endogenous killer T cells generated in normal B6 mice infected with PbA (**Figure 11A** **and data not shown**). This sequence also induced IFNγ production from endogenous T cells (**Figure 11B**) and PbT-I T cells (**Figure 11C**) responding to blood-stage infection. Note that tetramers made with K^b^ containing NCY were able to stain PbT-I cells, confirming the K^b^-restriction of this specificity (**data not shown**). The NCY peptide was derived from a protein of 745 amino acids (PBANKA_071450), which is now our leading candidate for the antigen responsible for priming PbT-I cells.

## Discussion

Here we characterize a new TCR transgenic mouse that produces CD8^+^ T cells specific for both the blood and liver stages of rodent malaria. PbT-I cells responded *in vivo* to the blood-stage of three different rodent *Plasmodium* species, PbA, *P. yoelii* XNL and *P. chabaudi* AS. In addition, PbT-I cells responded to mosquito-derived sporozoites of PbA and were able to provide protection against sporozoite infection. It remains to be tested as to whether PbT-I cells also recognize sporozoites from the other rodent *Plasmodium* species, but it seems likely that this will be the case given their blood-stage cross-reactivity. Recognition of blood-stage parasites as well as mosquito-derived sporozoites, and the ability to protect against liver-stage infection, suggests that the protein recognized by PbT-I cells is widely expressed throughout the parasite life cycle and is potentially well conserved. Identification of NCYDFNNI as a peptide recognized by PbT-I cells and by endogenous PbA-induced T cells suggests the protein encoded by PBANKA_071450, which is of unknown function and undefined expression pattern, may be the source of the PbT-I epitope. Construction of parasites deficient in this epitope will be required for formal proof.

It is notable that while the source protein is encoded in the genomes of *PbA* and *P. chabaudi*, the ortholog appears severely truncated in *P. yoelii* and consequently lacks the region containing NCYDFNNI found in other species. As PbT-1 cells were able to respond to *P. yoelii*, the authentic antigen must be present in this species. Whether this invalidates the gene product of PBANKA_071450 as the authentic PbT-I antigen, or is explained by sequencing error within the *P. yoelii* genome, or has some other basis remains to be established. Whatever the case, the NCYDFNNI epitope is clearly recognized by PbT-I cells and can be used to stimulate these transgenic T cells as well as endogenous T cells specific for PbA.

Evidence that immunization with live blood-stage parasites can protect against the liver-stage infection [25], suggests that multi-stage antigens like that recognized by PbT-I cells can be protective. Our study extends this concept by indicating that CD8^+^ T cells of a single specificity for a blood-stage antigen can protect against liver-stage infection when the antigen is also expressed during the liver stage. It has been reported that blood-stage infection can impair immunity to liver-stage antigens [24], though this is disputed by the above study, which uses blood-stage infection to induce anti-sporozoite immunity [25] and by another study that shows an equivalent response by liver-stage-specific transgenic T cells to sporozoites in the presence or absence of a subsequent blood-stage infection [32]. The availability of PbT-I cells will give us the opportunity to examine this relationship when the relevant antigen is expressed during both blood- and liver-stages and to determine how antigens presented during the blood-stage might influence the effector function of T cells capable of recognizing liver-stage antigens. Clearly, in our experiments, exposure of cells primed to liver-stage parasites did not impair their capacity to respond to blood-stage parasites, but increased the expansion of PbT-I cells. This raises the possibility that CD8^+^ T cells specific for antigens expressed in both stages of infection may have a selective advantage for expansion over single stage specific T cells.

The broad cross-reactivity of this TCR transgenic line means that it is suited to exploring the role of CD8^+^ T cells in several rodent malaria models. For blood-stage infection, this is most relevant to PbA, where ECM is dependent on CD8^+^ T cells. However, CD8^+^ T cells have been implicated in protective immunity to blood-stage infection by *P. yoelii 17XL* [35], raising the possibility that this protective process could be explored using PbT-I cells. These transgenic T cells should also be highly relevant for analysis of liver-stage immunity, as CD8^+^ T cells are critical for protection at this stage of infection [15], [36]. Here we used PbT-I cells to investigate the site of priming and T cell expansion after intravenous administration of irradiated sporozoites. This study was prompted by the implication that the effectiveness of this route of immunization was related to its capacity to prime in the liver [19]. Our analysis revealed that T cells showed signs of activation in the spleen and in the liver draining lymph nodes, but not in the liver itself, and subsequent examination of T cell proliferation showed that most PbT-I T cell proliferation occurred in the spleen. While our study does not exclude a role for the liver in tailoring the response, it suggests that at least the initial priming steps are unlikely to occur in this site. Thus, efficient priming via this route most likely derives from the large load of irradiated sporozoites deposited in the spleen after intravenous administration and the high frequency of T cells found in this organ. This contrasts infection by mosquito bite, which favors priming within skin draining lymph nodes [13], probably as a consequence of local deposition of sporozoites within the dermis of the skin. Our findings suggest that the spleen is the main site for priming sporozoite specific T cells after intravenous administration of parasites, but they do not formally exclude the liver draining lymph nodes or the liver as important sites of activation for protective immunity.

Initiation of PbT-I proliferation in the spleen in response to intravenous injection of irradiated PbA sporozoites also demonstrated that the sporozoites themselves expressed the antigen recognized by PbT-I cells and that conversion to later liver stages of development was not necessary to provide antigen capable of stimulating these T cells. Furthermore, it showed that the same DC subset as required for priming CD8^+^ T cell immunity to blood-stage infection, i.e the CD8α^+^ DC [28], [29], was responsible for inducing CD8^+^ T cell responses to the liver-stage parasites. Extraction of putative CD8α^+^ DC from the liver 6 days after sporozoite infection also suggested that these DC might contribute to antigen presentation in the liver at late time points after infection [37], though this idea should be taken with caution as CD8 T cells can express CD11c when activated and can be easily mistaken for DC. This common use of CD8α^+^ DC probably reflects their dominant capacity to cross-present antigens [38]. The ability of PbT-I cells to protect against infection by PbA sporozoites is encouraging because sterilizing immunity requires destruction of all infected hepatocytes. Our experiments used 7×10^6^ activated PbT-I cells to demonstrate protection, which is a relatively high number of cells but certainly achievable by vaccination. Identification of the antigen recognized by this TCR transgenic line should allow development of vaccination strategies to test the protective power of this potentially conserved antigen expressed in multiple stages of the life cycle. This approach has the potential to be highly effective since both stages of infection are shown to boost responses by CD8^+^ T cells with such multi-stage specificity. One concern with this type of multi-stage antigen, however, is that priming of T cells by sporozoites may enhance the potential for development of ECM mediated by the same cells during the blood-stage of the infection. While directly relevant for PbA infection where ECM is commonplace, this might not be of relevance to infection models where ECM is not seen e.g. *P. yoelii* XNL infection. Given the strongly argued lack of adaptive immune involvement in human cerebral malaria, this concern may also be irrelevant for human vaccination approaches. However, caution should be adopted here since our understanding of pathology in human cerebral malaria is still somewhat limited.

In conclusion, the PbT-I TCR transgenic line represents a versatile tool for studying CD8^+^ T cell immunity to a multitude of rodent *Plasmodium* species during both the liver- and blood-stages of infection. The current study highlights the spleen as a major organ of priming for intravenously-introduced blood- or liver-stage parasites and suggests that T cells with specificity for antigens expressed in both stages may contribute to pathology or protection, depending on the stage of life cycle.

## Methods

### Ethics statement

All procedures were performed in strict accordance with the recommendations of the Australian code of practice for the care and use of animals for scientific purposes. The protocols were approved by the Melbourne Health Research Animal Ethics Committee, University of Melbourne (ethic project IDs: 0810527, 0811055, 1112347, 0911527).

### Mice, mosquitos and parasites

C57BL/6 (B6) mice, B6.Ly5.1 mice, MHC I^-/-^ mice, K^b-/-^ mice, Batf3^-/-^ mice and the transgenic strains gBT-I [39] and PbT-I were used between 6-12 weeks and were bred and maintained at the Department of Microbiology and Immunology, The University of Melbourne. Batf3^-/-^ mice used in this study had been backcrossed 10 generations to B6. Animals used for the generation of the sporozoites were 4–5 week old male Swiss Webster mice were purchased from the Monash Animal Services (Melbourne, Victoria, Australia) and maintained at the School of Botany, The University of Melbourne, Australia.

*Anopheles stephensi* mosquitoes (strain STE2/MRA-128 from The Malaria Research and Reference Reagent Resource Center) were reared and infected with PbA as described [40]. Sporozoites were dissected from mosquito salivary glands [41], resuspended in cold PBS, irradiated with 20,000 rads using a gamma ^60^Co source, and administered to mice i.v. The rodent malaria lines PbA clone 15cy1, *P. chabaudi* AS and *P. yoelii* XNL were used in this study.

### Generation of transgenic PbT-I

Transgenic PbT-I mice were generated using the V(D)J segments of the TCRα- and β-genes of a CD8^+^ T cell hybridoma (termed B4) specific for an unidentified blood-stage PbA antigen. This hybridoma was derived from T cells extracted from the spleen of a B6 mouse at day 7 after infection with PbA. 3×10^6^ splenocytes from a mouse previously infected with PbA were co-cultured with 5×10^5^ conventional DC (extracted from the spleen of FMS-like tyrosine kinase 3 receptor ligand (Flt3-L) treated B6 mice) that were pre-loaded for 2 hours with 2×10^6^ PbA schizont lysate as previously described [42] in complete RPMI at 6.5% CO_2_, 37°C. One week later, cultured cells were re-stimulated for a week with fresh DC and PbA schizont lysate. To generate PbA-specific hybridomas, *in vitro* cultured cells were then fused with the BWZ36.GFP fusion partner and exposed to drug selection [43]. This led to isolation of the K^b^-restricted B4 hybridoma (Figure S1) from which PbT-I T cell receptor genes were derived.

TCR Vα usage was defined using 5′ RACE PCR on cDNA converted from the RNA of the B4 hybridoma. Sequencing analysis revealed that the TCR α-chain consisted of AV8S6 and Jα17 and Cα2 gene segments. The TCR α region was amplified by PCR from the cDNA of the B4 hybridoma using the forward primer GGATCC AGTGTCATTTCTTCCCT containing a *BamH*I recognition sequence at the 5′ end, designed to bind the 5′ UTR region of AV8S6, and the reverse primer CAGATCTCAACTGGACCACAG containing a *Bgl*II recognition sequence at the 5′ end, specific for the Cα region. The AV8S6-Jα17-Cα2 segment was cloned into the *BamH*I site of the pES4 cDNA expression vector, comprising the Ig-H chain enhancer, the H2-K^b^ promoter and the polyadenylation signal sequence of the human β-globulin gene [44]. To prepare the α-chain transgenic construct for microinjection, the pES4-VJC construct was digested with the restriction enzymes *Cla*I and *Not*I, and the digested mix was subjected to agarose gel electrophoresis. The ∼5.6 kb transgenic insert containing the VJC sequence, the promoter and enhancer sequences was excised from the gel, purified and quantitated for microinjection.

TCR Vβ usage was defined by PCR on cDNA converted from the RNA of the B4 hybridoma using the forward primer CCTGCCTCGAGCCAACTATGGG specific for the Vβ10 gene and the reverse primer CCAGAAGGTAGCAGAGACCC specific for the Cβ gene. Sequencing analysis revealed that the TCR β-chain consisted of Vβ10 (BV10S1A1), Dβ2 and Jβ2.2. The TCR β-chain was amplified by PCR from the genomic DNA of the B4 hybridoma using the forward primer GATCGATGTCCTAGGCCAGGAGATATGA specific for the Vβ10, incorporating a *Cla*I restriction enzyme site at the 5′ end, and the reverse primer GATCGATAAGCTCAGTCCAAGA specific for Jβ2.2 and incorporating a *Cla*I site at the 5′end. The Vβ10 (BV10S1A1), Dβ2 and Jβ2.2 segment was cloned into the *Cla*I site of the p3A9CβTCR gDNA expression vector, comprising the TCR β-chain enhancer, the 2B4-derived 5′ region and leader sequence and the B3-derived promoter and coding regions [45]. To prepare the construct for microinjection, the p3A9CβTCR VDJ construct was digested with the restriction enzymes *Apa*I and *Not*I, and the digested mix was subjected to agarose gel electrophoresis. The larger fragment (∼11 kb) transgenic insert containing the VDJ sequence, Cβ sequence and the promoter and enhancer sequences was excised from the gel, purified and quantitated for microinjection.

### Flow cytometry

Cells were labeled with monoclonal antibodies specific for CD8 (53-6.7), CD4 (RM 4-5), Thy1.2 (30-H12), CD45.1 (A20), Vα8.3 (B21.14), Vβ10 (B21.5) or CD69 (H1.2F3). Dead cells were excluded by propidium iodide staining. Cells were analyzed by flow cytometry on a FACsCanto or Fortessa (BD Biosciences), using the Flowjo software (Tree Star Inc.).

### Infection of mice and chloroquine treatment

Unless otherwise stated, mice were infected i.v. with 10^6^ PbA infected RBC (iRBC) in 0.2 ml of Hank's balanced salt solution (HBSS). In some experiments, mice were infected i.v. with 10^5^ *P.chabaudi* iRBC or i.v. with 10^4^ *P. yoelii* iRBC or with 300, 520, 900, 5×10^4^ or 10^5^ PbA sporozoites as stated in the figure legends. Mice infected with 10^4^ PbA infected RBC were injected i.p. with 0.4 mg chloroquine dissolved in water on days 6 and 7, before being euthanized for analysis on day 8 post-infection.

### T cell isolation and adoptive transfer

CD8^+^ T cells were negatively enriched from the spleens and lymph nodes of transgenic mice and labelled with CFSE as described [46]. Purified cells were injected i.v. in 0.2 ml HBSS. To deplete endogenous CD8^+^ T cells before adoptive transfer, B6 mice were injected i.p. with 100 µg of anti-CD8 antibody (clone 2.43) 7 days prior to the transfer of PbT-I or gBT-I cells.

### *In vivo* proliferation assay

1–2×10^6^ CFSE-labelled Ly5.1^+^ PbT-I cells were adoptively transferred into Ly5.2^+^ B6 mice a day before mice were infected with blood-stage PbA, *P. chabaudi,* or *P. yoelii* or with PbA sporozoites. In other experiments, 5×10^4^ or 1×10^6^ uGFP PbT-I cells labelled with CellTracker Violet stain (Invitrogen) were adoptively transferred into Ly5.2^+^ B6 or Batf3^-/-^ mice a day before infection with irradiated sporozoites, or 3 days before infection with PbA iRBC. Spleens and other organs were harvested on various days post-infection for the analysis of PbT-I proliferation by flow cytometry.

### Dendritic cell isolation

Dendritic cells were purified from the spleens of mice as previously described (28). Briefly, spleens were finely minced and digested in 1 mg/ml collagenase 3 (Worthington) and 20 µg/ml DNAse I (Roche) for 20 min at room temperature. After removing undigested fragments by filtering through a 70 µm mesh, cells were resuspended in 5 ml 1.077 g/cm^3^ nycodenz medium (Nycomed Pharma AS, Oslo, Norway), layered over 5 ml nycodenz medium and centrifuged at 1700×g at 4°C for 12 min. The light density fraction was collected and DC were negatively enriched by incubation with a cocktail of rat monoclonal anti-CD3 (clone KT3-1.1), anti-Thy-1 (clone T24/31.7), anti-Gr1 (clone RB68C5), anti-CD45R (clone RA36B2) and anti-erythrocyte (clone TER119) antibodies followed by immunomagnetic bead depletion using BioMag goat anti-rat IgG beads (Qiagen).

### Functional assay with hybridomas and IL-2 ELISA

5×10^4^ DC extracted from the spleens of naive WT, MHC-I-deficient or K^b^-deficient mice and resuspended in complete DMEM medium supplemented with 10% foetal calf serum (FCS) were cultured for 1 h with titrated amounts of lysed whole blood containing mixed stages of PbA parasites before adding 5×10^4^ B4 hybridoma cells. After culture for 40 h at 37°C in 6.5% CO_2_ supernatants were collected and concentrations of IL-2 were assessed using the Mouse IL-2 ELISA Ready-Set-Go kit (eBiosciences) following manufacturer's instructions.

### *In vitro* antigen presentation assay

PbA mixed blood-stages and schizont enriched parasite lysate were prepared as previously described [42]. Conventional DC isolated from the spleen of FMS-like tyrosine kinase 3 receptor ligand (Flt3-L) treated Ly5.2^+^ B6 mice [42] were incubated with titrated amounts of lysate from either the mixed blood-stages or the schizont-enriched parasites for 2 hours before the addition of CFSE-labeled Ly5.1^+^ PbT-I cells. After 60 hours of incubation at 6.5% CO_2_, 37°C, cells were harvested for analysis by flow cytometry.

### *Ex vivo* intracellular cytokine staining (ICS) assay

To detect degranulation and the production of cytokines IFNγ and TNFα from antigen specific cells, splenocytes from mice (either normal B6 mice or those adoptively transferred with PbT-I) infected for 7 days with irradiated sporozoites or 7–8 days with blood-stage PbA were restimulated by 5 µg/ml plate-bound anti-CD3 or 1 µg/ml peptide for 5 hours at 37°C in the presence of 10 µg/ml brefeldin A, monensin and anti-CD107 antibody (clone eBio1D4B). Cells were then surface labeled with antibodies and intracellular cytokine staining was performed to detect intracellular IFNγ and TNFα using Cytofix/Cytoperm Fixation and Permeabilization Solution (BD) according to the manufacturer's instructions. Results were represented in Venn diagrams using the online tool at www.venndiagram.tk.

### Generation of *in vitro* activated CTL

PbT-I or gBT-I isolated from the spleen and lymph nodes were stimulated with media containing 10% FCS, 10 U/ml IL-2 and 5 µg/ml anti-CD28 in 75 cm^2^ tissue culture flasks pre-coated with 10 µg/ml anti-CD3 (clone 2c11), anti-CD8 (clone 53-6.7) and anti-CD11a (clone 121/7.7). 40 hours later, cell cultures were divided into two equal volumes and given an equivalent volume of fresh medium before culturing for 24 hours. Cells were then harvested and centrifuged over lymphocyte separation media to remove dead cells. *In vitro* activated cells generated using this method were routinely >90% pure.

### Isolation of cells from the brain

Mice were perfused intracardially with 10 ml PBS prior to harvesting of the brain. Brains were cut into fine fragments, washed once with media and digested with collagenase/DNAse (1 mg/ml collagenase III (Roche); 20 µg/ml DNAse I, (Worthington) for 1 hour at room temperature with rotation. Samples were filtered through 75 µm nylon mesh to remove undigested fragments and then centrifuged once at 596 g, 5 minutes at 4°C. The pellet was resuspended in 7 ml 33% Percoll diluted in media, and centrifuged at 400 g for 20 minutes at room temperature with low brake. The supernatant was discarded and the pellet containing RBC was incubated with 500 µl RBC lysis buffer for 2 minutes on ice. Cells were washed twice with FACS buffer followed by surface staining with various antibodies.

### Generation and monitoring of ECM

Mice infected with blood-stage PbA were monitored daily for the development of ECM. Mice were considered to have ECM when showing signs of neurological symptoms such as ataxia and paralysis, evaluated as the inability of mice to self-right.

### Hematoxilin and eosin (H&E) staining of brain sections

Brains were fixed in 4% paraformaldehyde followed by 70% ethanol overnight and then stained by H&E.

### Combinatorial peptide library scan

An octamer combinatorial peptide library in positional scanning format [34] was synthesized (Pepscan Presto, Netherlands). For combinatorial peptide library screening, splenocytes from transgenic PbT-I mice were purified, washed and rested overnight in RPMI 1640 containing 100 U/ml penicillin, 100 µg/ml streptomycin, 2 mM L-glutamine and 2% heat inactivated fetal calf serum (all Life Technologies). In 96-well U-bottom plates, 6×10^4^ splenocytes target cells were incubated with 160 library mixtures (at 100 µM) in duplicate for two hours at 37°C. Following peptide pulsing, 3×10^4^ PbT-I splenocytes were added and the assay was incubated overnight at 37°C. The supernatant was then harvested and assayed for MIP-1β by ELISA according to the manufacturer's instructions (R&D Systems).

### Assessment of peptide stimulation of PbT-I cells by CD69 expression

5×10^5^ GFP-expressing PbT-I lymph node and spleen cells together with 10^5^ B6 spleen cells and peptide (titrated in 10-fold steps from 5–5000 pM) were pelleted together in a 96-well U-bottom plate and incubated for 3 hours at 37°C (6.5% CO_2_). Cells were then stained with antibodies specific for CD8 and CD69 and the proportion of CD69^+^CD8^+^GFP^+^ cells determined.

### *In vivo* CTL assay for lytic activity

To detect peptide-specific lytic activity *in vivo*, mice were infected for 7 days with 10^6^ blood-stage PbA and cured by chloroquine treatment from day 4–6 before adoptive transfer of target cell populations. *In vivo* cytotoxicity was performed essentially as described [42], with the modification that target cells were a mixture of CFSE^lo^ B6 spleen cells, DsRed-expressing splenocytes and GFP-expressing splenocytes, the latter two populations coated with test peptides at 1 µg/ml. Equal numbers of cells were combined and 2.4×10^7^ cells were injected into host mice and 18 h later spleen cells were harvest for flow cytometric assessment of lysis 18 h later within the spleen.

## Supporting Information

Figure S1 **K^b^ restricted recognition by the B4 hybridoma specific for PbA.** Dendritic cells were enriched from the spleens of naive B6 (filled circle), MHC-I-deficient (filled triagle), or K^b^-deficient (open diamond) mice and cultured for 1 h with titrated amounts of lysed blood-stage PbA. B4 hybridoma cells (from which the PbT-I TCR genes were isolated) were then added to the cultures for 40 h before measuring IL-2 in the supernatant by ELISA. Data points denote mean of IL-2 concentration and error bars represent SEM. Data were pooled from 2 independent experiments.
(PDF)

Figure S2 **Characterization of T cells from the lymph node of PbT-I mice.** Cells were harvested from the lymph nodes of PbT-I transgenic or littermate control B6 mice (WT). FACS analysis was performed to characterize the expression of CD8, CD4 and the transgenic TCR alpha (Vα8.3) and beta (Vβ10) chains. Representative histograms show the expression of the transgenic TCR Vα8.3 and Vβ10 chains on the CD8 (upper) and CD4 (lower) single-positive cells from the LN. This experiment was repeated three times with two mice per experiment.
(PDF)

Figure S3 **Enumeration of T cells in the spleen, lymph nodes and thymus of PbT-I mice.** Cells were harvested from the spleen, lymph nodes or thymus of PbT-I transgenic or littermate control wild-type (WT) mice. (A) The total number of live cells and (B) the proportion of T cells expressing either CD4 or CD8 for the spleen and lymph nodes or CD4 or CD8 or double positive (DP) for the thymus. This experiment was repeated three times with two mice per experiment.
(PDF)

Figure S4 **Characterization of cells in the thymus of PbT-I mice.** (A) Representative dot-plots showing CD4 and CD8 expression in the thymus of PbT-I mice or littermate WT controls. (B) Representative histograms showing the expression of the transgenic TCR Vα8.3 and Vβ10 chains on the single positive CD8 or CD4, double positive (DP) and double negative (DN) thymocytes. This experiment was repeated three times with two mice per experiment.
(PDF)

Figure S5 **PbT-I is specific for blood-stage PbA.** (A) 10^5^ CFSE labeled PbT-I cells were incubated with 2×10^5^ dendritic cells that were pre-incubated with titrated amounts of PbA lysate from either schizonts-enriched (filled circle) or mixed blood-stage parasites (open circle). 60 hours later, the proliferation of PbT-I cells was assessed by flow cytometry. Data are pooled from two experiments.
(PDF)

Figure S6 **PbT-I T cells do not respond to herpes simplex virus type 1 infection.** B6 mice were adoptively transferred with 10^6^ Ly5.1 gBT-I cells together with 10^6^ GFP-expressing PbT-I cells, each population labeled with Cell-Tracker Violet. The next day, mice were infected i.v. with 10^4^ blood-stage PbA or 10^6^ pfu HSV-1 or were left uninfected (Naïve). Spleens were harvested five days later and the proliferation of PbT-I and gBT-I cells was analyzed. (A) Representative histograms showing the proliferation of PbT-I cells and gBT-I cells in naïve mice or on day five post-infection. Note that PbT-I cells have a natural higher level of homeostatic proliferation than gBT-I cells, as shown in naïve hosts. (B) Number of PbT-I cells (left) or gBT-I cells (right) in the spleen of naïve mice or those infected with either blood-stage PbA or HSV-1 for five days. Data shown from one of two representative experiments.
(PDF)

Figure S7 **PbT-I cells primed during a blood-stage PbA infection are functionally competent.** B6 mice were adoptively transferred with 5×10^4^ GFP-expressing PbT-I cells and the next day infected i.v. with 10^4^ blood-stage PbA. Infected mice were injected i.p. with 0.4 mg chloroquine on days 6 and 7 to cure of parasitemia. Eight days after infection, spleens were harvested and intracellular cytokine staining was performed to assess degranulation (CD107a) and cytokine production (IFNγ and TNFα) by PbT-I cells. (A) Bar graph showing the mean percentage of PbT-I cells expressing CD107a, IFNγ or TNFα. Error bars represent standard error of the mean. Data are pooled from two experiments with two mice per experiment. (B) Venn diagram depicting the co-expression of cytokines and CD107a by PbT-I cells from a representative mouse.
(PDF)

Figure S8 **Accumulation of CD8^+^ T cells in the brains of mice given PbT-I cells and infected with blood-stage PbA.** Mice adoptively transferred with PbT-I cells (filled circle) or gBT-I cells (filled square) or no cells (open circle) were sacrificed on days 4, 5 or 6 post-infection with blood-stage PbA and their brains were analyzed for the infiltration of CD8^+^ T cells. Total number of CD8^+^ T cells sequestered in the brains of mice at the times shown. Data are pooled from 2-4 experiments. Data were compared using student t test (*, p<0.05).
(PDF)

Figure S9 **PbT-I cells induced ECM after PbA infection.** Hematoxilin and eosin staining of sagittal sections of the brains of PbA-infected C57BL/6 mice. Mice were divided into three cohorts and either left untreated (A–C), depleted of endogenous CD8 T cells (D–F), or transferred with 2×10^6^ naïve PbT-I cells 7 days after endogenous CD8 T cell depletion (G–I). One day after PbT-I transfer mice were infected with 10^6^ blood-stage PbA. On day 6 after infection, untreated and PbT-I transferred mice developed ECM. All mice were then killed and their brains removed for histological examination. Typical leukocyte and RBC aggregates could be found in the brain vessels (A, G) and meninges surrounding cerebellar folia (C, I) of ECM-developing mice. These were absent from ECM-resistant mice (D, F). The olfactory bulbs of mice with ECM showed widespread haemorrhages (B, H), in contrast with their ECM-resistant counterparts (E). Size bars: 50 µm.
(PDF)

Figure S10 **PbT-I cells cross-react with** ***P. yoelii*** **XNL.** B6 mice were adoptively transferred i.v. with 5×10^5^ CFSE-labeled PbT-I. The next day, mice were injected i.p. with 10^5^ *P. yoelii* XNL. Six days later, spleens were harvested and the proliferation of PbT-I was analyzed. The lines represent the mean and each data point represents a mouse. Data are from one representative experiment of two. Data were compared using student t test (**, p<0.01).
(PDF)

Figure S11 **B6 mice were adoptively transferred with 5×10^4^ GFP-expressing PbT-I and the next day infected i.v. with 10^5^ irradiated sporozoites.** Eight days later, spleens were harvested and intracellular cytokine staining was performed to assess degranulation (CD107a) and cytokine production (IFNγ and TNFα) by PbT-I cells. (A) Percentage of PbT-I cells expressing CD107a, IFNγ or TNFα. Error bars represent standard error of the mean. Data are pooled from two experiments with four mice per group. (B) Venn diagram depicting the co-expression of cytokines and CD107a by PbT-I cells from a representative mouse.
(PDF)

Figure S12 **The effect of inflammation on expansion of PbT-I cells to irradiated sporozoites.** (A) PbT-I T cells proliferate to sporozoite antigen when introduced 2 days but not 7 days after injection of irradiated sporozoites. B6 mice were untreated (naïve, grey lines) or injected with 5×10^4^ irradiated sporozoites (RAS, black lines) on day 0 and then 0, 2 or 7 days later were transferred with 2.5×10^5^ CellTracker Violet-labeled GFP-expressing PbT-I cells. On day 7 after PbT-I cell transfer, spleens were harvested and the proliferation profile of PbT-I cells examined. Data are representative of 1-3 experiments. (B) Number of PbT-I cells recovered from mice shown in (A). Closed symbols are from 1-3 experiments with 2.5×10^5^ transferred PbT-I cells. Open symbols are from 2 experiments with 5.0×10^4^ transferred PbT-I cells. Each time point represents at least 3 experiments. Data were log10 transformed and compared by one-way ANOVA and Tukey's multiple comparison test. (***, p<0.001, n.s. p>0.05). (C) CpG oligonucleotide induced inflammation did not enhance expansion of PbT-I cells. B6 mice were adoptively transferred with 5 x 10^4^ GFP-expressing PbT-I cells and left uninfected (naïve) or infected with 5×10^4^ irradiated sporozoites (RAS). Two days later, mice were left untreated or injected with 20 nmol of 1668 CpG oligonucleotide (+ CpG). On day 7 spleens were harvested and PbT-I cells enumerated. Data are pooled from two experiments. Data were compared by one-way ANOVA and Tukey's multiple comparison test. There were no significant differences between similar groups treated with or without CpG (p>0.05).
(PDF)

Figure S13 **To identify Ly5.1^+^ PbT-I cells by flow cytometry after adoptive transfer into B6 mice, cells from tissues of recipient mice were gated sequentially as shown in graphs A–E. PbT-I cells were identified as Ly5.1^+^, Vα8.3^+^ CD8^+^ cells.**
(PDF)
