# Supplementary figures and images for: CD8+ T Cells from a Novel T Cell Receptor Transgenic Mouse Induce Liver-Stage Immunity That Can Be Boosted by Blood-Stage Infection in Rodent Malaria
Source: PLoS Pathog. 2014 May 22;10(5):e1004135. doi: 10.1371/journal.ppat.1004135 (PMC4031232; doi:10.1371/journal.ppat.1004135)

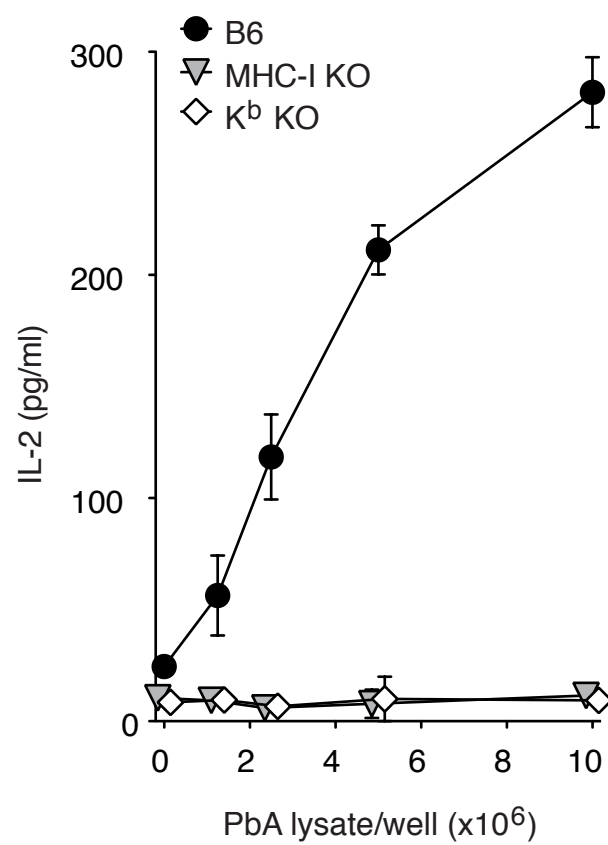

Suppl Figure 1

Supplement: Figure S1 — Kb restricted recognition by the B4 hybridoma specific for PbA. Dendritic cells were enriched from the spleens of naive B6 (filled circle), MHC-I-deficient (filled triagle), or Kb-deficient (open diamond) mice and cultured for 1 h with titrated amounts of lysed blood-stage PbA. B4 hybridoma cells (from which the PbT-I TCR genes were isolated) were then added to the cultures for 40 h before measuring IL-2 in the supernatant by ELISA. Data points denote mean of IL-2 concentration and error bars represent SEM. Data were pooled from 2 independent experiments. (PDF) [file ppat.1004135.s001.pdf]

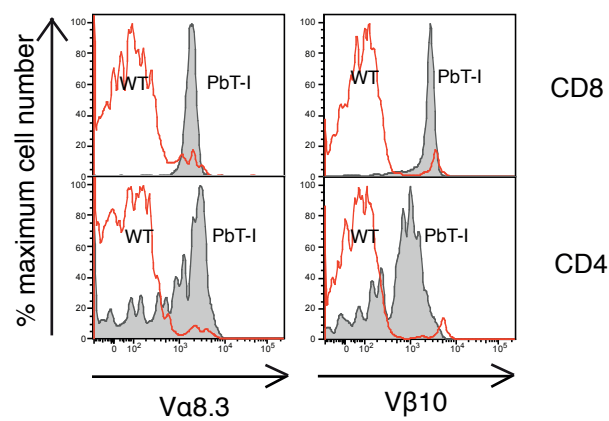

Suppl Figure 2

Supplement: Figure S2 — Characterization of T cells from the lymph node of PbT-I mice. Cells were harvested from the lymph nodes of PbT-I transgenic or littermate control B6 mice (WT). FACS analysis was performed to characterize the expression of CD8, CD4 and the transgenic TCR alpha (Vα8.3) and beta (Vβ10) chains. Representative histograms show the expression of the transgenic TCR Vα8.3 and Vβ10 chains on the CD8 (upper) and CD4 (lower) single-positive cells from the LN. This experiment was repeated three times with two mice per experiment. (PDF) [file ppat.1004135.s002.pdf]

**A**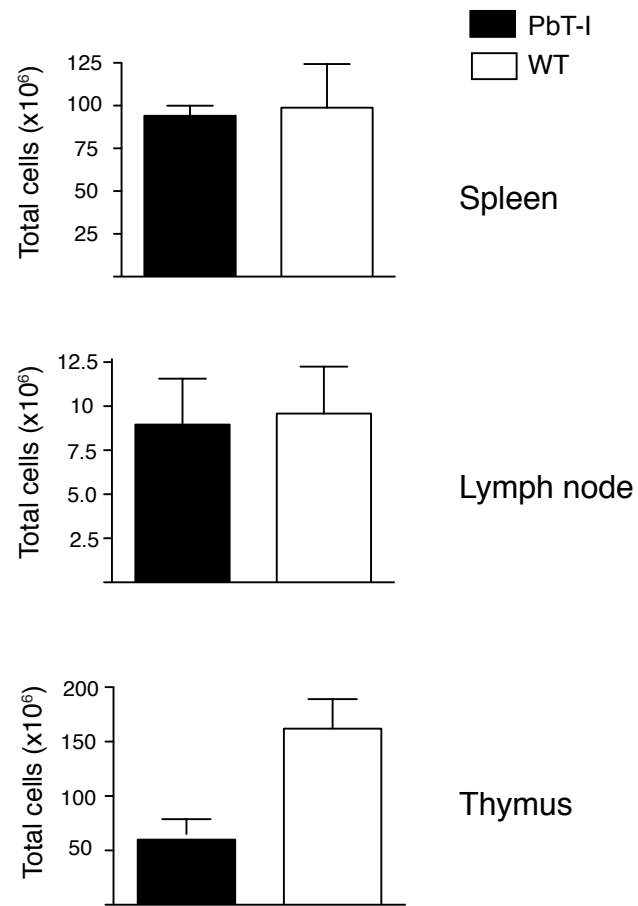**B**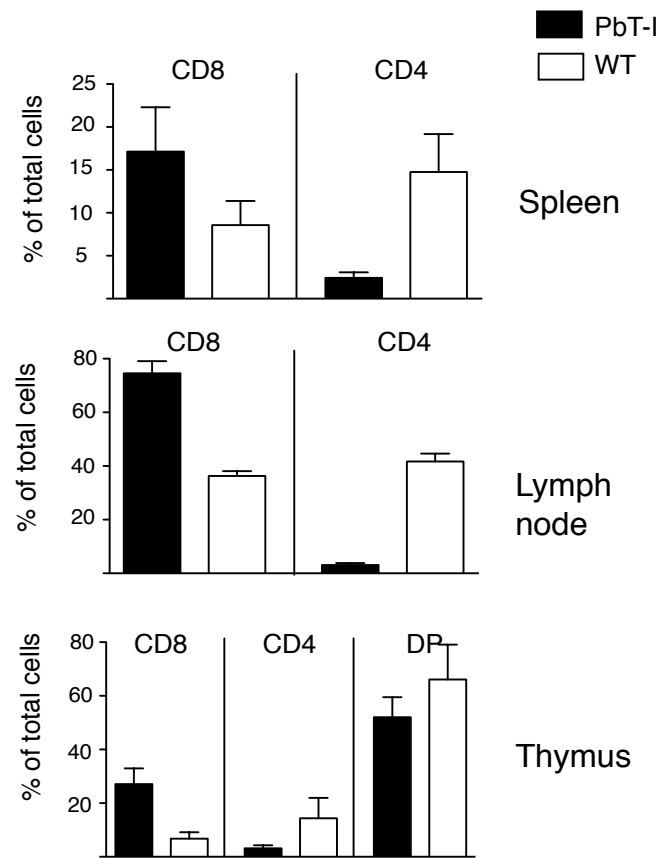

Supplement: Figure S3 — Enumeration of T cells in the spleen, lymph nodes and thymus of PbT-I mice. Cells were harvested from the spleen, lymph nodes or thymus of PbT-I transgenic or littermate control wild-type (WT) mice. (A) The total number of live cells and (B) the proportion of T cells expressing either CD4 or CD8 for the spleen and lymph nodes or CD4 or CD8 or double positive (DP) for the thymus. This experiment was repeated three times with two mice per experiment. (PDF) [file ppat.1004135.s003.pdf]

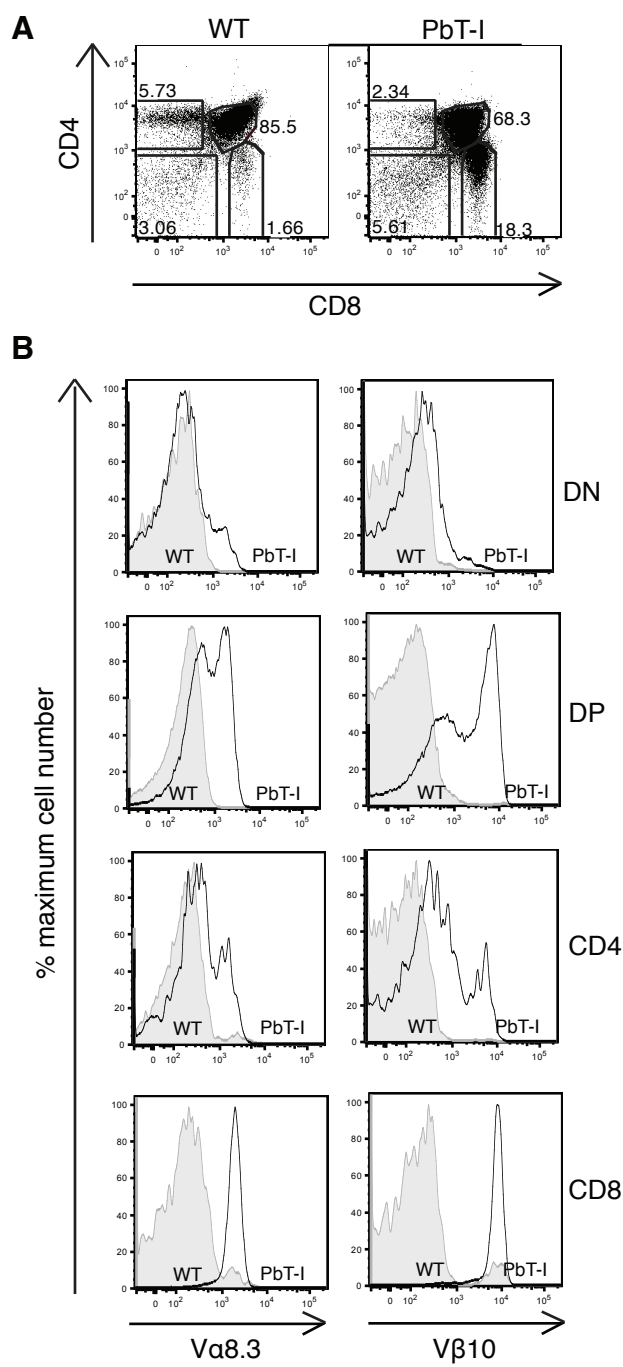

Suppl Figure 4

Supplement: Figure S4 — Characterization of cells in the thymus of PbT-I mice. (A) Representative dot-plots showing CD4 and CD8 expression in the thymus of PbT-I mice or littermate WT controls. (B) Representative histograms showing the expression of the transgenic TCR Vα8.3 and Vβ10 chains on the single positive CD8 or CD4, double positive (DP) and double negative (DN) thymocytes. This experiment was repeated three times with two mice per experiment. (PDF) [file ppat.1004135.s004.pdf]

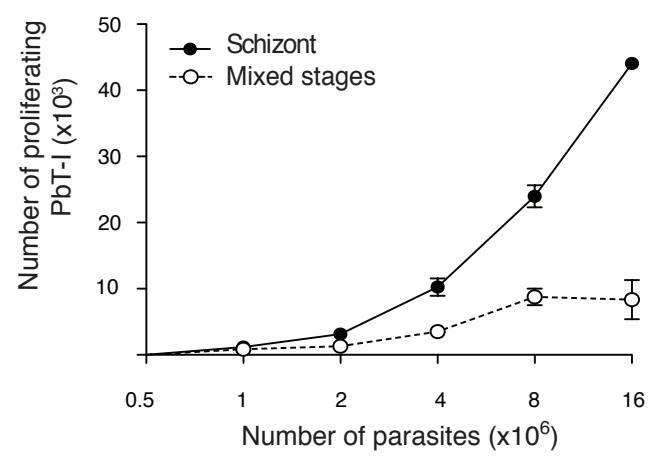

Suppl Figure 5

Supplement: Figure S5 — PbT-I is specific for blood-stage PbA. (A) 105 CFSE labeled PbT-I cells were incubated with 2×10^5^ dendritic cells that were pre-incubated with titrated amounts of PbA lysate from either schizonts-enriched (filled circle) or mixed blood-stage parasites (open circle). 60 hours later, the proliferation of PbT-I cells was assessed by flow cytometry. Data are pooled from two experiments. (PDF) [file ppat.1004135.s005.pdf]

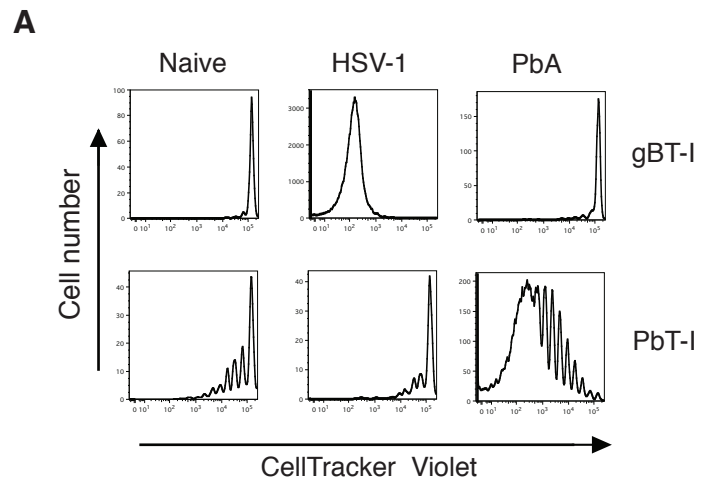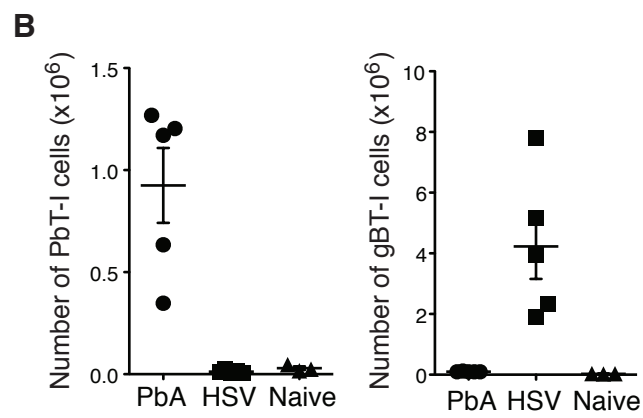

Supplement: Figure S6 — PbT-I T cells do not respond to herpes simplex virus type 1 infection. B6 mice were adoptively transferred with 106 Ly5.1 gBT-I cells together with 106 GFP-expressing PbT-I cells, each population labeled with Cell-Tracker Violet. The next day, mice were infected i.v. with 104 blood-stage PbA or 106 pfu HSV-1 or were left uninfected (Naïve). Spleens were harvested five days later and the proliferation of PbT-I and gBT-I cells was analyzed. (A) Representative histograms showing the proliferation of PbT-I cells and gBT-I cells in naïve mice or on day five post-infection. Note that PbT-I cells have a natural higher level of homeostatic proliferation than gBT-I cells, as shown in naïve hosts. (B) Number of PbT-I cells (left) or gBT-I cells (right) in the spleen of naïve mice or those infected with either blood-stage PbA or HSV-1 for five days. Data shown from one of two representative experiments. (PDF) [file ppat.1004135.s006.pdf]

**A**

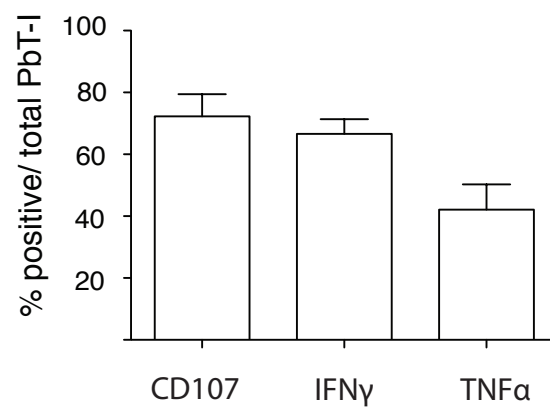

**B**

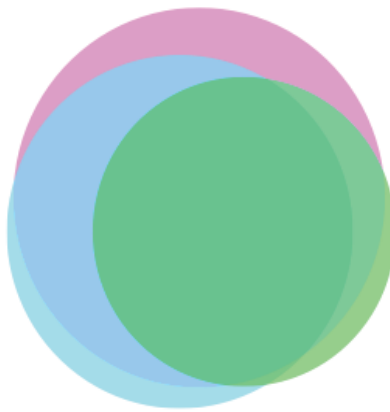

pink - CD107a - 80.9%  
blue - IFN $\gamma$  - 70.1%  
green - TNF $\alpha$  - 53.7%

Supplement: Figure S7 — PbT-I cells primed during a blood-stage PbA infection are functionally competent. B6 mice were adoptively transferred with 5×10^4^ GFP-expressing PbT-I cells and the next day infected i.v. with 104 blood-stage PbA. Infected mice were injected i.p. with 0.4 mg chloroquine on days 6 and 7 to cure of parasitemia. Eight days after infection, spleens were harvested and intracellular cytokine staining was performed to assess degranulation (CD107a) and cytokine production (IFNγ and TNFα) by PbT-I cells. (A) Bar graph showing the mean percentage of PbT-I cells expressing CD107a, IFNγ or TNFα. Error bars represent standard error of the mean. Data are pooled from two experiments with two mice per experiment. (B) Venn diagram depicting the co-expression of cytokines and CD107a by PbT-I cells from a representative mouse. (PDF) [file ppat.1004135.s007.pdf]

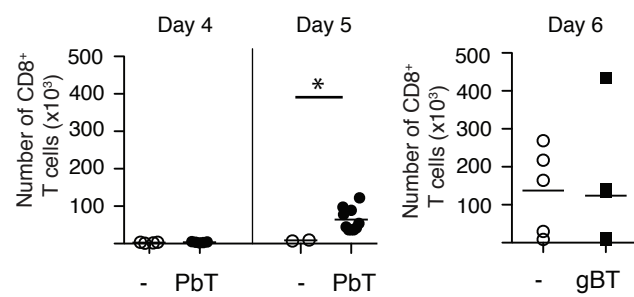

Suppl Figure 8

Supplement: Figure S8 — Accumulation of CD8+ T cells in the brains of mice given PbT-I cells and infected with blood-stage PbA. Mice adoptively transferred with PbT-I cells (filled circle) or gBT-I cells (filled square) or no cells (open circle) were sacrificed on days 4, 5 or 6 post-infection with blood-stage PbA and their brains were analyzed for the infiltration of CD8+ T cells. Total number of CD8+ T cells sequestered in the brains of mice at the times shown. Data are pooled from 2-4 experiments. Data were compared using student t test (*, p<0.05). (PDF) [file ppat.1004135.s008.pdf]

**B6**

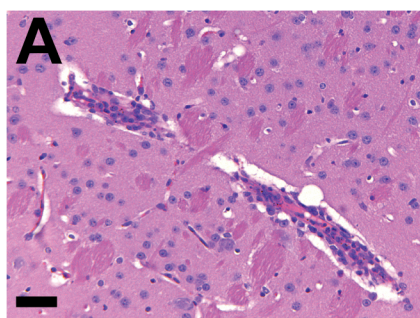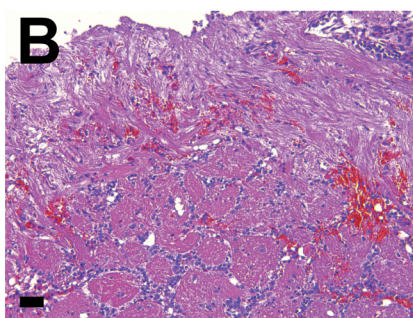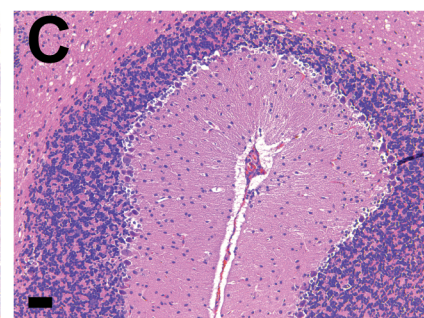

**B6, -CD8**

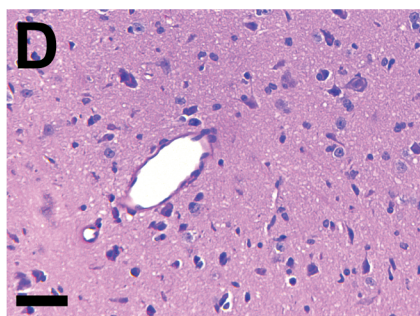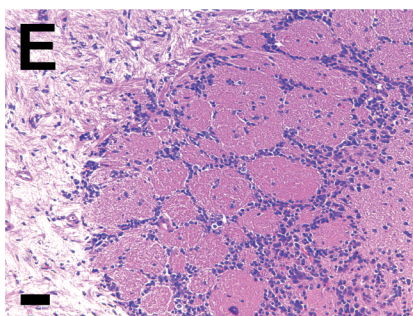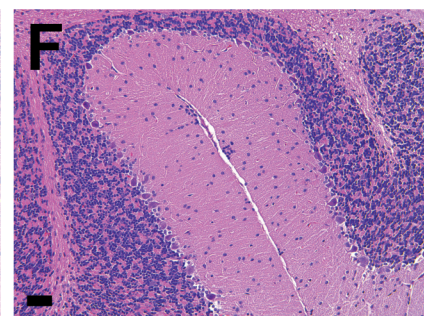

**B6, -CD8,  
+PbT-I**

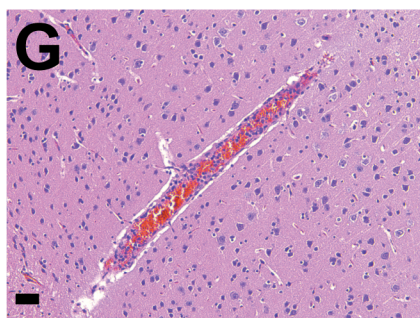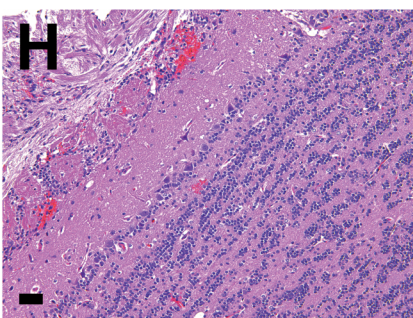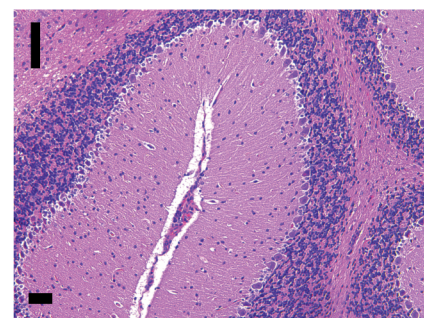

Supplement: Figure S9 — PbT-I cells induced ECM after PbA infection. Hematoxilin and eosin staining of sagittal sections of the brains of PbA-infected C57BL/6 mice. Mice were divided into three cohorts and either left untreated (A–C), depleted of endogenous CD8 T cells (D–F), or transferred with 2×10^6^ naïve PbT-I cells 7 days after endogenous CD8 T cell depletion (G–I). One day after PbT-I transfer mice were infected with 106 blood-stage PbA. On day 6 after infection, untreated and PbT-I transferred mice developed ECM. All mice were then killed and their brains removed for histological examination. Typical leukocyte and RBC aggregates could be found in the brain vessels (A, G) and meninges surrounding cerebellar folia (C, I) of ECM-developing mice. These were absent from ECM-resistant mice (D, F). The olfactory bulbs of mice with ECM showed widespread haemorrhages (B, H), in contrast with their ECM-resistant counterparts (E). Size bars: 50 µm. (PDF) [file ppat.1004135.s009.pdf]

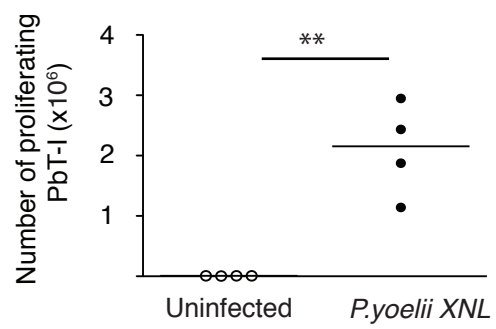

Supplement: Figure S10 — PbT-I cells cross-react with P. yoelii XNL. B6 mice were adoptively transferred i.v. with 5×10^5^ CFSE-labeled PbT-I. The next day, mice were injected i.p. with 105 P. yoelii XNL. Six days later, spleens were harvested and the proliferation of PbT-I was analyzed. The lines represent the mean and each data point represents a mouse. Data are from one representative experiment of two. Data were compared using student t test (**, p<0.01). (PDF) [file ppat.1004135.s010.pdf]

**A**

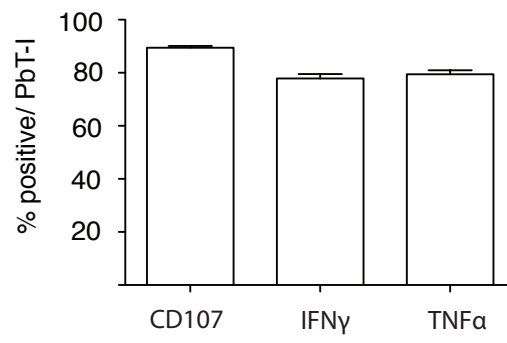

**B**

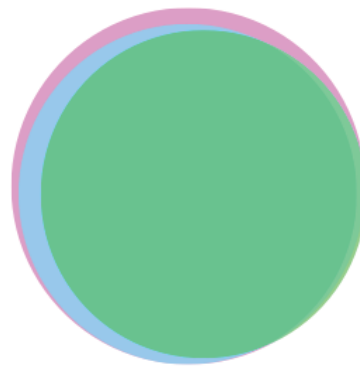

pink - CD107a - 88.4%  
blue - IFN $\gamma$  - 80.6%  
green - TNF $\alpha$  - 74.8%

Supplement: Figure S11 — B6 mice were adoptively transferred with 5×10^4^ GFP-expressing PbT-I and the next day infected i.v. with 105 irradiated sporozoites. Eight days later, spleens were harvested and intracellular cytokine staining was performed to assess degranulation (CD107a) and cytokine production (IFNγ and TNFα) by PbT-I cells. (A) Percentage of PbT-I cells expressing CD107a, IFNγ or TNFα. Error bars represent standard error of the mean. Data are pooled from two experiments with four mice per group. (B) Venn diagram depicting the co-expression of cytokines and CD107a by PbT-I cells from a representative mouse. (PDF) [file ppat.1004135.s011.pdf]

**A**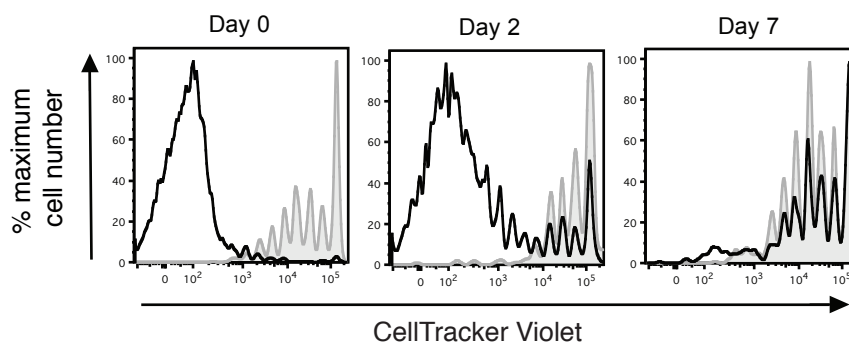**B**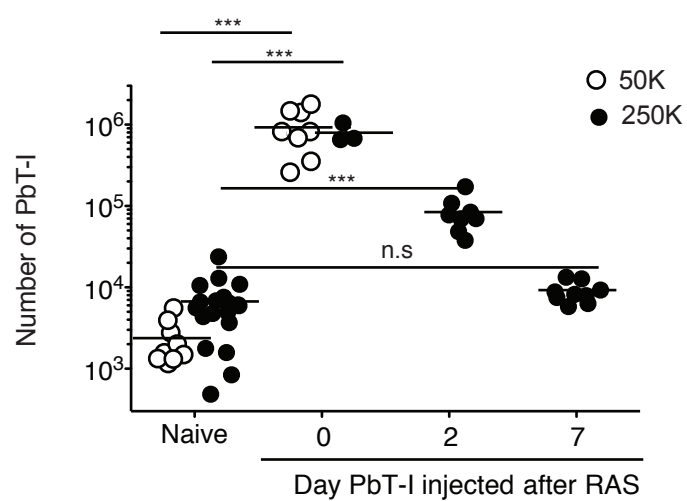**C**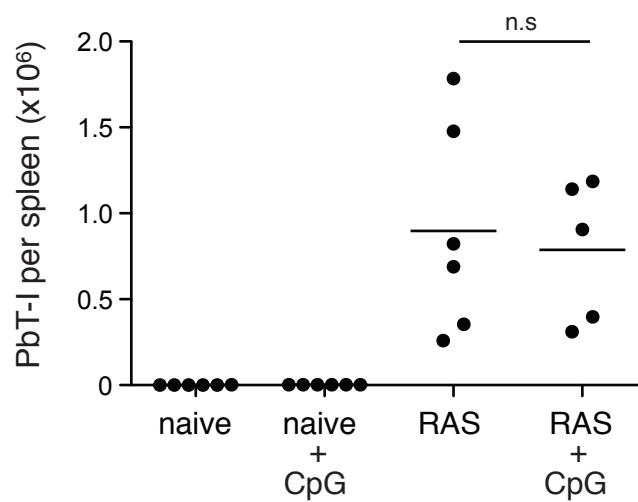

Supplement: Figure S12 — The effect of inflammation on expansion of PbT-I cells to irradiated sporozoites. (A) PbT-I T cells proliferate to sporozoite antigen when introduced 2 days but not 7 days after injection of irradiated sporozoites. B6 mice were untreated (naïve, grey lines) or injected with 5×10^4^ irradiated sporozoites (RAS, black lines) on day 0 and then 0, 2 or 7 days later were transferred with 2.5×10^5^ CellTracker Violet-labeled GFP-expressing PbT-I cells. On day 7 after PbT-I cell transfer, spleens were harvested and the proliferation profile of PbT-I cells examined. Data are representative of 1-3 experiments. (B) Number of PbT-I cells recovered from mice shown in (A). Closed symbols are from 1-3 experiments with 2.5×10^5^ transferred PbT-I cells. Open symbols are from 2 experiments with 5.0×10^4^ transferred PbT-I cells. Each time point represents at least 3 experiments. Data were log10 transformed and compared by one-way ANOVA and Tukey's multiple comparison test. (***, p<0.001, n.s. p>0.05). (C) CpG oligonucleotide induced inflammation did not enhance expansion of PbT-I cells. B6 mice were adoptively transferred with 5 x 10^4^ GFP-expressing PbT-I cells and left uninfected (naïve) or infected with 5×10^4^ irradiated sporozoites (RAS). Two days later, mice were left untreated or injected with 20 nmol of 1668 CpG oligonucleotide (+ CpG). On day 7 spleens were harvested and PbT-I cells enumerated. Data are pooled from two experiments. Data were compared by one-way ANOVA and Tukey's multiple comparison test. There were no significant differences between similar groups treated with or without CpG (p>0.05). (PDF) [file ppat.1004135.s012.pdf]

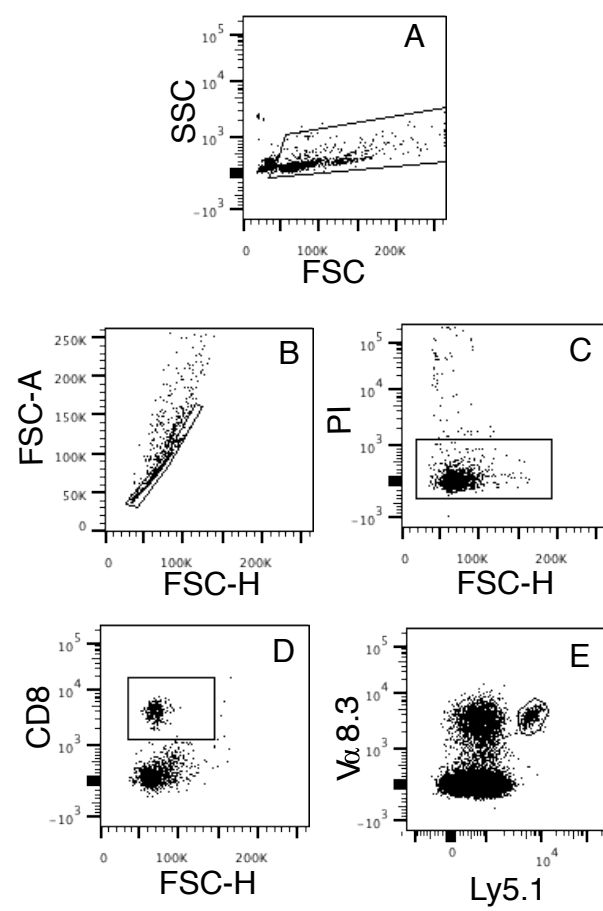

Supplement: Figure S13 — To identify Ly5.1+ PbT-I cells by flow cytometry after adoptive transfer into B6 mice, cells from tissues of recipient mice were gated sequentially as shown in graphs A–E. PbT-I cells were identified as Ly5.1+, Vα8.3+ CD8+ cells. (PDF) [file ppat.1004135.s013.pdf]
